# Genome‐Wide DNA Methylation Patterns Predict Age in the Zebra Shark (*Stegostoma tigrinum*) and Provide Insight Into the Evolution of Vertebrate Aging

**DOI:** 10.1111/mec.70326

**Published:** 2026-04-03

**Authors:** Samantha L. Bock, Kady Lyons, Lei Yang, Jennifer Wyffels, Lance Adams, Nienke Klerks, Aaron Jeskie, Ellen Leever, Taylor Hartl, Javier Almunia, Dan Peterson, Ana Ferreira, Joe Okamoto, Kate Archibald, Chris Spaulding, Kayla Leyden, Gisele A. Montano, Savannah Vasey, Adrienne Rowland, Lise Watson, Alfonso Lopez, Jay Hemdal, Mike Stafford, Anne Bronikowski, Gavin J. P. Naylor, Benjamin B. Parrott

**Affiliations:** ^1^ Department of Integrative Biology, W. K. Kellogg Biological Station Michigan State University Hickory Corners Michigan USA; ^2^ Savannah River Ecology Laboratory University of Georgia Aiken South Carolina USA; ^3^ Georgia Aquarium Atlanta Georgia USA; ^4^ Department of Marine Sciences University of Georgia Athens Georgia USA; ^5^ Florida Museum of Natural History Gainesville Florida USA; ^6^ Marine Science Research Center Ripley's Aquariums Myrtle Beach South Carolina USA; ^7^ Delaware Biotechnology Institute University of Delaware Newark Delaware USA; ^8^ Aquarium of the Pacific Long Beach California USA; ^9^ Burgers' Zoo Arnhem the Netherlands; ^10^ Columbus Zoo and Aquarium Powell Ohio USA; ^11^ Life Sciences Department Golden Nugget Las Vegas Nevada USA; ^12^ Jenkinson's Aquarium Point Pleasant Beach New Jersey USA; ^13^ Loro Parque Fundación Tenerife Spain; ^14^ Minnesota Zoo Apple Valley Minnesota USA; ^15^ Oceanario de Lisboa Lisboa Portugal; ^16^ Okinawa Churaumi Aquarium, Okinawa Churashima Foundation Okinawa Japan; ^17^ Omaha's Henry Doorly Zoo and Aquarium Omaha Nebraska USA; ^18^ Point Defiance Zoo and Aquarium Tacoma Washington USA; ^19^ SEA LIFE Kansas City Aquarium Kansas City Missouri USA; ^20^ SeaWorld Parks Orlando Florida USA; ^21^ SEA LIFE Carlsbad Aquarium Carlsbad California USA; ^22^ Shark Reef Aquarium at Mandalay Bay Las Vegas Nevada USA; ^23^ John G. Shedd Aquarium Chicago Illinois USA; ^24^ Singapore Oceanarium at Resorts World Sentosa Sentosa Island Singapore; ^25^ Toledo Zoo and Aquarium Toledo Ohio USA; ^26^ Wonders of Wildlife National Museum and Aquarium Springfield Missouri USA; ^27^ Odum School of Ecology University of Georgia Athens Georgia USA

**Keywords:** aging, DNA methylation, epigenetic clock, shark

## Abstract

Epigenomic changes are a hallmark of aging, and DNA methylation (DNAm) has emerged as the most reliable molecular marker of an individual's age. Genome‐wide patterns of age‐associated hypo‐ and hypermethylation have been applied to generate predictive models (i.e., “epigenetic clocks”) capable of estimating chronological age in an increasingly diverse set of species including many mammals, a few birds, a reptile, and several bony fishes. Elasmobranchs (sharks, skates, and rays) are underrepresented in comparative investigations of epigenetic aging despite exhibiting exceptional life history variation, occupying a key basal position in the vertebrate phylogeny, and encompassing a large proportion of threatened species lacking accurate, non‐lethal age determination methods. Here, we characterize epigenome‐wide aging signals in the zebra shark (*Stegostoma tigrinum*), a long‐lived elasmobranch of conservation concern, from whole‐genome enzymatic methyl‐sequencing of whole blood. Using a cohort of 51 known‐age aquarium‐bred individuals, we develop several epigenetic clock models capable of predicting chronological age with a median absolute error of 1.03–1.99 years (3.32%–6.42% of lifespan) based on the methylation status of as few as ten cytosines. We further apply our models to 19 individuals of unknown age originating from the wild. By profiling the broader age‐associated methylome we demonstrate that these patterns not only predict age with high accuracy but also exhibit striking similarities in their genomic distributions to those observed in mammals pointing to conservation of the processes underlying epigenetic aging across vertebrates.

## 1 Introduction

All organisms undergo some form of aging. Yet despite the near universality of biological aging, species exhibit considerable intra‐ and interspecific variation in their lifespans and aging trajectories (Jones et al. 2014; Ma and Gladyshev 2017). Advances in understanding the molecular and evolutionary drivers of variation in aging across diverse species has been limited by a lack of analytical tools to quantify age, especially in long‐lived taxa (Nussey et al. 2013; Reinke et al. 2022). DNA methylation (DNAm), the covalent addition of a methyl group to a cytosine base typically within the context of a cytosine‐phosphate‐guanine dinucleotide (“CpG”), has emerged as one of the most reliable molecular markers of age (Horvath and Raj 2018; Seale et al. 2022). Age‐associated patterns of hypo‐ and hyper‐methylation across the genome can be used to generate predictive models (i.e., “epigenetic clocks”) estimating chronological age with high accuracy based on the methylation status of a discrete set of loci (Horvath and Raj 2018). One of the first human epigenetic clocks developed in 2013 predicted chronological age within 3.6 years based on 353 sites (Horvath 2013). Since then, epigenetic clocks have been developed for a wide range of mammals (Anderson et al. 2021; Bors et al. 2021; Horvath et al. 2022; Lu, Fei, et al. 2023; Polanowski et al. 2014; Stubbs et al. 2017; Thompson et al. 2017; Wilkinson et al. 2021; Zoller et al. 2024), a few birds (de Paoli‐Iseppi et al. 2019; Haller et al. 2025; Raddatz et al. 2021), one reptile (Shealy et al. 2025), two amphibians (Zoller et al. 2024), and several bony fishes (Anastasiadi and Piferrer 2020; Bertucci et al. 2021; Mayne et al. 2020; Weber et al. 2022; Weber, Fields, et al. 2024) opening new opportunities for comparative investigations into age‐specific life history variation (Parrott and Bertucci 2019) and contributing practical conservation tools to quantify age structure in wild populations (de Paoli‐Iseppi et al. 2019; Jarman et al. 2015; Piferrer and Anastasiadi 2023).

Mounting evidence suggests age‐associated DNAm patterns are more than passive biomarkers and may be causally linked to age‐related functional decline (Yang et al. 2023). In humans and some mammalian model organisms, deviations of DNAm‐based “epigenetic age” from chronological age are associated with the timing of life history transitions (e.g., menarche, menopause; Binder et al. 2018; Levine et al. 2016), disease risk (Levine et al. 2015, 2018; Lu et al. 2019), mortality (Marioni et al. 2015), and environmental factors that affect longevity (Maegawa et al. 2017; Petkovich et al. 2017; Ryan et al. 2020; Zannas et al. 2015). In addition, rates of change in methylation at conserved age‐associated sites exhibit a consistent negative relationship with lifespan across mammal species (Lowe et al. 2018). Researchers have even developed a “pan‐mammalian” epigenetic clock capable of predicting age in samples from 185 mammal species and 59 tissue types (Lu, Fei, et al. 2023). Taken together, epigenetic aging signals appear to represent a highly conserved feature of biological aging in mammals with direct links to age‐associated functional decline. However, the extent to which these findings extend across the vertebrate tree of life remains unclear.

Epigenetic clocks developed in non‐mammalian taxa provide a promising basis for the hypothesis that epigenetic aging signals are at least partially conserved across vertebrates; however, varied DNAm profiling methods and limited taxonomic representation obscures insights into the functional significance of these patterns and evolutionary forces shaping them (Tangili et al. 2023; Zoller et al. 2024). Many studies of epigenetic aging in non‐mammalian taxa have implemented targeted, reduced‐representation, or reference genome‐free approaches to measure DNAm (e.g., multiplex PCR with bisulfite sequencing, bisulfite‐converted restriction site‐associated DNA sequencing, bsRADseq; Tangili et al. 2023). While these approaches provide benefits in terms of the reduced cost and resources required to develop an epigenetic clock, they confer less information about the dynamics of the broader aging methylome and genomic context (i.e., proximity to genes, regulatory regions, etc.) in which these patterns occur. This is a critical knowledge gap as methylation patterns occurring in different regions of the genome can have vastly different functional consequences. For example, regions of high CpG density, termed “CpG islands”, tend to be lowly methylated in vertebrates, but changes in the methylation status of these regions in proximity to genes can alter gene activity (Weber et al. 2007). Further, the density of CpGs in a region is linked to its gene regulatory potential (M. Weber et al. 2007). Importantly, in mammals, age‐associated methylation changes occurring in different genomic contexts (e.g., CpG density, proximity to genes) vary in their direction, rate, and level of interspecific conservation (Lu, Fei, et al. 2023; Moqri et al. 2025). In particular, age‐associated loss of methylation tends to occur in gene‐poor regions of the genome with lower CpG density, while age‐associated increases in methylation tend to occur in regions of high CpG density (e.g., CpG islands and surrounding regions termed CpG shores and shelves) that are bound by specific transcription factors (Moqri et al. 2025). Whether these patterns of genomic localization hold outside of mammals is largely unknown, thereby impeding our understanding of the evolution of epigenetic aging more broadly. Further, key vertebrate lineages are underrepresented or absent from comparative investigations of epigenetic aging.

Chondrichthyans, encompassing elasmobranchs (sharks, skates, rays) and chimaeras, are one such group for which data on epigenetic aging patterns are largely absent. Yet this group occupies a critical phylogenetic position diverging from the rest of vertebrates over 400 million years ago (Benton et al. 2009; Inoue et al. 2010) and exhibits exceptional life history diversity (Frisk et al. 2001). For example, within sharks alone, lifespan varies from approximately 10 years in the Atlantic sharpnose (*Rhizoprionodon terranovae*; Branstetter 1987) and 17 years in the bonnethead ( *Sphyrna tiburo* ; Frazier et al. 2014) to over 70 years in the white shark ( *Carcharodon carcharias* ; Hamady et al. 2014) and possibly over 272 years in the Greenland shark ( *Somniosus microcephalus* ; Nielsen et al. 2016). Beyond the features making chondrichthyans a particularly informative clade for comparative aging research, this group also encompasses a large proportion of threatened and endangered species (Dulvy et al. 2021) lacking reliable, non‐lethal markers of age (Cailliet 2015). Population age structures can provide key insights into population growth dynamics and have important consequences for population viability analysis (Boyce 1992; Jackson et al. 2020), yet age structures of threatened chondrichthyan populations are rarely measured. Current methods of age determination in chondrichthyans involve counting vertebral rings; however, these methods require lethal sampling and are subject to a high degree of error, especially in older individuals (Natanson et al. 2018). Epigenetic clocks capable of predicting age from blood or other non‐lethally sampled tissues could therefore provide valuable tools for conservation efforts.

Here we profile genome‐wide patterns of DNAm in a known‐aged cohort of zebra sharks (*Stegostoma tigrinum*), an elasmobranch of conservation concern and popular aquarium species (Dudgeon et al. 2008, 2009; Rigby et al. 2024), across the entire lifespan, ranging from neonates (< 1 year) to individuals nearing the species' maximum lifespan (> 28 years) (Ebert et al. 2021). With our data, we sought to answer the following questions: (1) To what extent does the DNA methylome change with age in an elasmobranch? (2) How are age‐associated DNAm patterns distributed across the genome? (3) How accurately can DNAm patterns at a subset of loci predict age in an elasmobranch? Recent work in the lemon shark ( *Negaprion brevirostris* ) and cownose ray ( *Rhinoptera bonasus* ) provided promising preliminary evidence for the existence of age‐associated DNAm patterns in elasmobranchs; however, both species currently lack reference genomes (Beal et al. 2022; Weber, Wyffels, et al. 2024). In the present study, we leverage the zebra shark's high‐quality reference genome (Lee et al. 2025; Rhie et al. 2021) and aquarium breeding programs to characterize broad age‐associated DNAm patterns and the genomic context in which they occur. We further develop multiple epigenetic clock models capable of predicting age with high accuracy in aquarium‐bred individuals and apply these models to predict age in individuals originating from the wild. Our findings not only demonstrate striking similarities in the genomic localization of epigenetic aging signals across vertebrates but also raise important considerations for future efforts to develop epigenetic clocks using aquarium or zoo specimens.

## 2 Materials and Methods

### 2.1 Study Animals and Sample Curation

**FIGURE 1:**
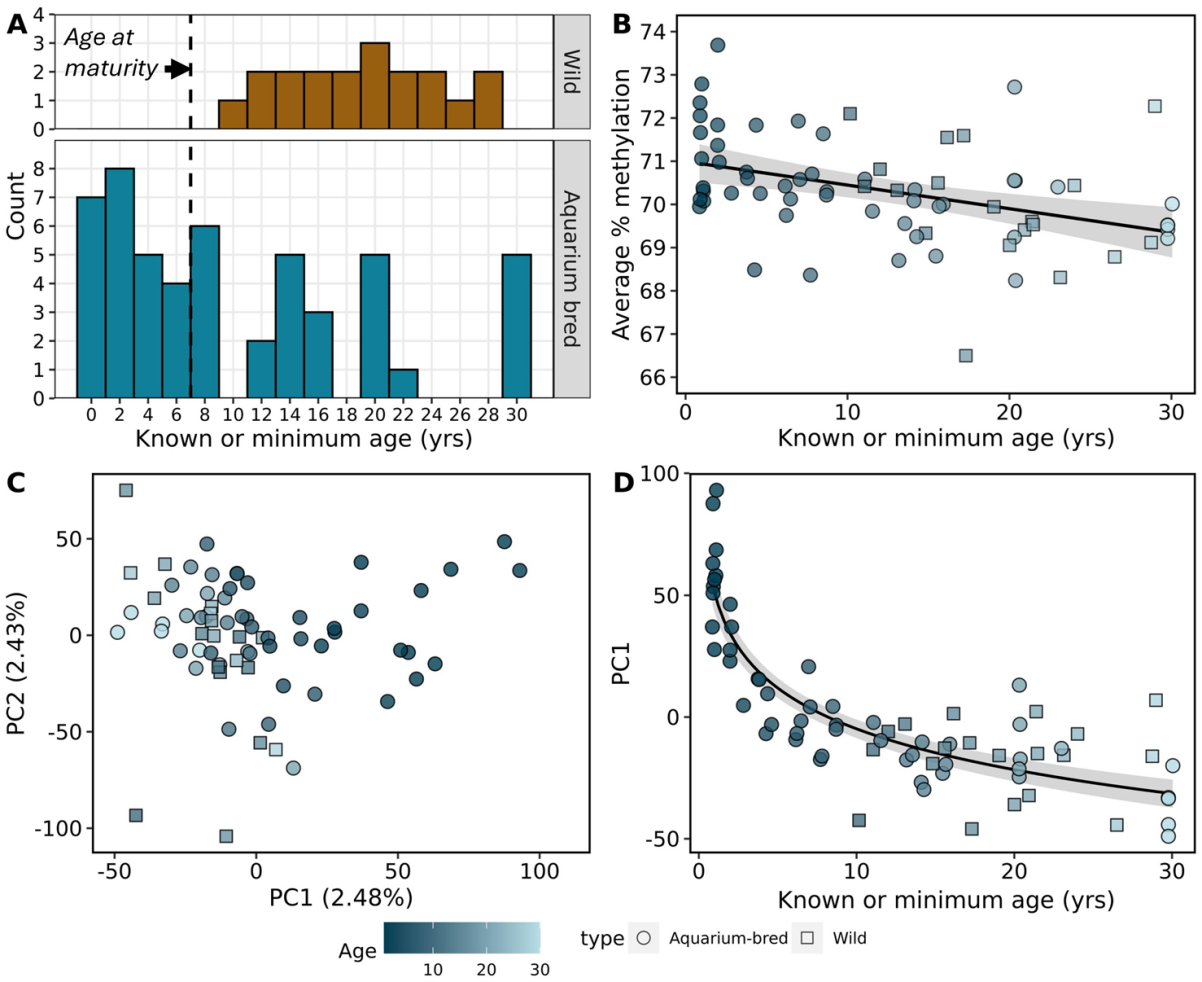
Sampling design and broad‐scale DNA methylation patterns. (A) Histogram depicting distribution of aquarium‐bred (*n* = 51) and wild‐caught (*n* = 19) zebra shark whole blood (or red blood cell) samples across the life span. Chronological age was precisely known for aquarium‐bred sharks and minimum age was known for wild‐caught sharks based on time since aquarium acquisition. (B) Genome‐wide average percent methylation across all filtered CpGs exhibited a significant negative relationship with known and minimum age. (C) Principal component analysis based on the methylation status of all filtered CpGs did not indicate the presence of any samples with aberrant methylation profiles. (D) The first principal component (PC1) was strongly correlated with age. Line depicts a polynomial regression of PC1 by known or minimum age for all samples.

Whole blood samples taken from zebra sharks ranging in age from 0 to 30 years old were collected from aquarium partners in accordance with institutional standards and national laws during routine veterinarian examinations. The oldest individuals in our study are among the oldest known zebra sharks in aquaria, with this species' maximum lifespan reported as > 28 years (Ebert et al. 2021). Chronological age was known for individuals born in aquariums according to institutional records of hatch date. Minimum age, but not chronological age, was known for wild‐caught individuals based on time since initial aquarium acquisition (Figure 1A). Whole blood (or isolated red blood cells for two samples) was preserved in RNAlater, ethanol, or without any preservative and stored at—20°C until DNA extraction via a modified column approach based on the Promega SV RNA extraction protocol (Bae et al. 2021). DNA quality was assessed using a NanoDrop spectrometer (Thermo Fisher Scientific Inc., Waltham, MA, USA) and DNA concentration was determined with a Qubit fluorometer (Thermo Fisher Scientific Inc., Waltham, MA, USA). Samples were selected for subsequent library preparation and sequencing based on prioritization of accurate known‐ages or age estimates, high‐quality genomic DNA (gDNA), bias towards the youngest and oldest animals, and balance of sex ratio. Target sample size was guided by previous benchmarking studies which recommend a minimum calibration population of ~70 (Mayne et al. 2021). Samples from 70 individuals were selected for sequencing: 41 females and 29 males ranging from 0.9 to 30 years old (Figure 1A). Of these samples, 51 individuals were aquarium‐bred (AB) and 19 individuals were wild‐caught (WC).

### 2.2 Library Preparation and Sequencing

Libraries were prepared from 200 ng of gDNA using the New England Biolabs (Ipswich, MA, USA) NEBNext Enzymatic Methyl‐Seq (EM‐Seq) kit (Catalogue #E720S) according to manufacturer instructions. Unmethylated lambda phage and methylated pUC19 control sequences were spiked into each library. Prior to proceeding with library preparation, gDNA and spike‐in sequences were sheared to a target peak fragment size of 300 bp using a Covaris M220 Focused‐ultrasonicator (Covaris Inc., Woburn, MA, USA). Preliminary sequencing of seven libraries was performed at high coverage (~26.9× ±3.4; mean ±1 SD) on an Illumina NovaSeq 6000 with S4 reagents, generating an average 535.3 million paired‐end reads (150 bp) per sample. Down‐sampling of high coverage samples was performed to estimate required sequencing effort to achieve target coverage for subsequent libraries. Library preparation of the remaining 63 samples was performed in four batches and sequencing was performed across eight runs on either the Illumina NovaSeq 6000 S4 or NovaSeq X Plus by the University of Florida Interdisciplinary Center for Biotechnology Research, generating an average of 186.2 million paired‐end reads (150 bp) per sample (Table S1).

### 2.3 Quality Control, Read Alignment, and Methylation Data Processing

Raw sequencing reads were assessed for quality with FastQC (v. 0.11.9; https://www.bioinformatics.babraham.ac.uk/projects/fastqc/) and MultiQC (v. 1.14; Ewels et al. 2016), then trimmed with TrimGalore! (v. 0.6.7; https://www.bioinformatics.babraham.ac.uk/projects/trim_galore/) to remove adapter sequences and low‐quality reads (“‐stringency 1 –clip_r1 4 –clip_r2 4 –three_prime_clip_r1 4 –three_prime_clip_r2 4”; Figure S1). Alignment of trimmed reads to the most recent version of the zebra shark genome (sSteTig4; GCF_030684315.1) and methylation calling was performed with Bismark (v. 0.24.1; Krueger and Andrews 2011) and Bowtie2 (v. 2.4.5; Langmead and Salzberg 2012). Average alignment rates were 79.6% (±4.2% SD). Overall average percent methylation was 68.9% in a CpG context, 0.48% in a CHG context, and 0.39% in a CHH context. Trimmed reads were also aligned to control lambda phage and pUC19 sequences (as supplied by NEB). Conversion efficiency was 99.6% (±0.2% SD) based on unmethylated lambda and 98.2% (±3.1% SD) based on methylated pUC19.

Alignment files in BAM format were deduplicated with Bismark and sorted and indexed with Samtools (v. 1.16.1; Li et al. 2009). Following deduplication, samples exhibited a mean genomic coverage of 11.1X (±5.6 SD). Sorted BAM files were read into R (v. 4.2.1; R Core Team 2022) with the “processBismarkAln” function in the methylKit package (v. 1.24.0; Akalin et al. 2012). Analyses were limited to methylation occurring in a CpG context, as methylation in CHG and CHH contexts was minimal for zebra sharks and is generally rare in vertebrates (Feng et al. 2010). Using methylKit and GenomicRanges packages (v. 1.50.2; Lawrence et al. 2013), reads on both strands of a CpG site were merged and loci were filtered to remove those in the 99.9th percentile of coverage. Loci were further filtered to only include those covered by at least 5 reads in 80% of samples (*n* = 56). Read coverage was normalized between samples using a scaling factor based on differences in the median of coverage distributions with the “normalizeCoverage” function in methylKit. Unbiased clustering of samples based on the methylation status of all filtered sites (*n* = 14,807,085 CpGs) was performed via principal components analysis.

### 2.4 Identification of Age‐Associated DNAm Patterns

Age‐associated loci were identified by assessing Spearman and Pearson correlations between the methylation status of individual loci and chronological age in AB animals using the “corAndPvalue” function within the WGCNA package (v. 1.73; Langfelder and Horvath 2008). To correct for the false discovery rate (FDR) associated with multiple comparisons, a Benjamini–Hochberg correction was applied to *p*‐values. We defined age‐associated sites as loci exhibiting an absolute correlation coefficient > 0.5 and FDR < 0.05.

As a complementary approach to this correlation analysis, we also performed differential methylation analysis between individuals defined as “young” (age ≤ 6.2 years; prior to sexual maturity (Ebert et al. 2021); *n* = 21), “mid” (6.2 years < age ≤ 14.2 years; post‐maturity adults; *n* = 15), and “old” (age > 14.2 years; older adults; *n* = 15) using logistic regression with correction for overdispersion according to McCullagh and Nelder (1989) in methylKit (“calculateDiffMeth(overdispersion = ‘MN’)”). Categorial age groups were defined based on reproductive maturity status and the distribution of ages in our dataset. Prior to differential methylation analysis, we further filtered out constitutively hypermethylated and hypomethylated sites (CpGs with percent methylation > 90% or < 10% across all samples) and invariant sites using the “nearZeroVar” function in the caret package (v. 6.0–92; Kuhn 2008; post‐variance filtering *n* = 14,293,069). Loci were considered differentially methylated according to FDR < 0.05. This categorical approach allowed us to define broader patterns of age‐associated methylation trajectories including those that change in contrasting directions over the lifespan. Loci that were significantly differentially methylated between the “young” and “mid” group or “mid” and “old” group were further characterized into one of four categories based on the direction of methylation difference in each contrast. Loci were characterized as “negative–negative” if they were hypomethylated in the older group in both contrasts, “negative–positive” if they were hypomethylated with age earlier in life and hypermethylated with age later in life, “positive–negative” if they were hypermethylated with age early in life and hypomethylated with age later in life, and “positive–positive” if they were hypermethylated in the older group in both contrasts.

### 2.5 Annotation of Age‐Associated DNAm Patterns

All covered, filtered CpGs were annotated according to their gene context (promoter, exon, intron, or intergenic), CpG island context (island, shore, shelf, or open sea) and distance to the closest transcriptional start site (TSS) using the GenomicRanges package. Annotations for NCBI RefSeq genes and gene predictions as well as CpG islands were downloaded in BED format from the UCSC Genome Browser's Table Browser tool (Perez et al. 2024; https://genome.ucsc.edu). From the CpG island coordinates, locations of shores (±2000 bp of islands) and shelves (±2000 bp of shores) were defined using BEDTools (v. 2.30.0; Quinlan and Hall 2010). Each covered, filtered CpG was annotated according to gene context and CpG island context using the “countOverlaps” function in the GenomicRanges package. If a locus overlapped multiple gene contexts, it was defined according to the following precedent: promoter > exon > intron > intergenic. The distance to and identity of the closest TSS of each CpG was determined using the “getAssociationWithTSS” function in the GenomicRanges package. Two‐sided Fisher's exact tests were used to assess whether loci of interest were over‐ or underrepresented in each of the annotation categories.

Gene ontology (GO) term overrepresentation analysis was performed with gene identities of TSSs closest to CpGs of interest using the clusterProfiler package (v. 4.12.6; Wu et al. 2021; Yu et al. 2012). To allow for use of GO annotations assigned to *S. tigrinum* genes through the NCBI Eukaryotic Genome Annotation Pipeline, an organism‐specific annotation database was constructed using the “makeOrgPackageFromNCBI” function in the AnnotationForge package (v. 1.40.2; Carlson and Pagès 2025). GO overrepresentation analysis was then performed for each annotation category (“biological process”, “cellular component”, and “molecular function”) against a custom background of genes closest to all filtered CpGs with the “enrichGO” function in clusterProfiler. Terms with an FDR < 0.05 were considered significantly enriched.

Genes in proximity to CpG sites displaying age‐associated methylation patterns were also characterized according to the transcription factors (TFs) that target them using Enrichr (Chen et al. 2013; Kuleshov et al. 2016) with the ChEA Transcription Factor Targets 2022 database (Keenan et al. 2019; Lachmann et al. 2010). We limited this analysis to genes with age‐associated CpGs within 5 kb of their TSS. Human orthologs of CpG‐proximate genes were identified using the orthogene package with the “gprofiler” method (v. 1.10.0; Schilder and Skene 2022). Genes for which a one‐to‐one human ortholog could not be identified were excluded from this analysis. Of the 20,539 unique genes with a filtered CpG within 5 kb of its TSS, we identified human orthologs for 8136 genes (39.6%). Genes in proximity to age‐associated loci were compared against a custom background of all unique genes with TSSs within 5 kb of any filtered CpG. TF associations with an FDR < 0.05 were considered significantly enriched.

### 2.6 Epigenetic Clock Calibration and Validation

Three approaches were taken to calibrate the DNAm‐based age predictor. First, we used a leave‐one‐out cross validation (LOOCV) approach in which 51 models were trained on 50 of the 51 AB samples and tested on the single sample left out of the training set. Second, we trained a single model on 41 AB individuals (~80%) and held out the remaining 10 AB individuals (~20%) as a test set. Third, we trained a model using all 51 AB samples and used the WC samples as an entirely naïve test set. The machine‐learning algorithm used to calibrate the epigenetic age predictor does not tolerate missing data (Zou and Hastie 2005). Therefore, prior to clock calibration, percent methylation values were imputed at the sample level for sites with missing values (i.e., locus without sufficient read coverage in a sample) using a *k*‐nearest neighbour approach (*k* = 5) with the “impute.knn” function in the impute package (v. 1.72.3; Troyanskaya et al. 2001). For each of the calibration approaches, elastic net regularized regression was implemented to fit the model using the glmnet package (v. 4.1‐4; Friedman and Hastie 2010; Zou and Hastie 2005). Elastic net regularization applies a penalty to model beta coefficients such that coefficients of uninformative CpGs are reduced to zero and thereby removed from the model. The model relies on two hyperparameters, alpha which controls the balance between ridge and lasso regularization and lambda which controls the strength of the penalty on model beta coefficients (Zou and Hastie 2005). Alpha was set to 0.5 after testing multiple values via a LOOCV approach with the AB samples (Appendix S1). This is also the default value for elastic net regression and aligns with methods used to calibrate DNAm‐based age predictors in other species (Bertucci et al. 2021; Horvath 2013; Petkovich et al. 2017). Lambda was selected via 5‐fold internal cross‐validation for each calibration approach based on the value that minimized mean squared error (Zou and Hastie 2005). Models were fit using raw chronological age (in years). We also tested the effect of assigning higher observation weights to older animals in order to improve prediction performance in older samples. Specifically, mature individuals (> 6.2 years) were assigned an observation weight of 2 and immature individuals were assigned an observation weight of 1.

As a complement to the elastic net regularized regression, we also identified the top 10 and 20 age‐associated sites according to the Pearson correlation coefficient in each of the test sets and fit a simple linear regression of age based on the methylation status of those sites. In all cases, model performance was assessed based on the correlation (Pearson's *r*) and median absolute difference (i.e., error) between actual age and DNAm‐based predicted age of both the training and test sets.

### 2.7 Assessment of Clock Performance on Wild‐Caught Individuals

Chronological age was not known for individuals caught in the wild and subsequently brought into aquariums, though minimum age (time since acquisition) was known. This presented a challenge with respect to assessing model performance because a discrepancy between estimated age and DNAm‐predicted age could arise for multiple reasons including wild‐caught individuals being acquired by aquariums at advanced ages, a difference in the performance of the model on aquarium‐bred versus wild‐caught individuals, or a true difference in the epigenetic aging trajectories of aquarium‐bred versus wild‐caught individuals. To address the first source of error and improve minimum age estimates for wild‐caught individuals, we used information from a subset of these individuals for which total length was measured at or after acquisition to estimate age at acquisition. To do this, we constructed a von Bertalanffy growth curve from repeated total length data that was opportunistically collected during routine health assessments. Data on total length at ages ranging from 0 to 7 years for 43 aquarium‐bred individuals (*n* = 285 total measurements) was modelled using a non‐linear mixed effects model in the nlme package (v. 3.1–168; Pinheiro et al. 2025) according to the typical parameterization of the von Bertalanffy growth equation (Equation (1); Ogle 2013).

(1) $$E\left[L|t\right]=L_{\infty }\left(1-e^{-K\left(t-t_{0}\right)}\right)$$

where $E\left[L|t\right]$ is length at time *t*, $L_{\infty }$ is asymptotic length, *K* is the Brody growth rate coefficient, and *t*_*0*_ is a modelling artefact representing the age when length is zero. To account for repeated measures, we included random intercepts for each individual ID. Fit statistics of the non‐linear mixed effects model were: log‐likelihood = −882.44 and AIC = 1784.88. The inverse of the von Bertalanffy growth equation was then used with the fixed effect parameters only ($L_{\infty }$ = 264.4 cm, *K* = 1.022 × 10^−3^, *t*_*0*_ = −113.3) to predict age given the lengths of a subset of wild‐caught individuals measured at or after aquarium acquisition (*n* = 10; Figure S5). The length of time between acquisition and measurement was subtracted from estimated age at measurement to yield estimated age at acquisition which was then used to generate a corrected minimum age. There are several caveats associated with this approach to estimating age at acquisition from data on total length. For one, we constructed the growth curve from a relatively small sample of young aquarium‐bred zebra sharks. Importantly, growth trajectories of individuals hatched in aquariums may differ from those of individuals caught in the wild. However, it is more likely that we have underestimated age at acquisition than overestimated it given growth rates are expected to be higher in early life and in aquariums where dietary resources are not limited. Thus, the estimated ages at acquisition can serve as conservative corrections to the minimum ages previously based solely on time since acquisition.

### 2.8 Identification of Sex‐Associated DNAm Patterns

Sex‐associated variation in DNAm patterns has been observed across a range of taxa (Bock et al. 2022; Gatev et al. 2021; Rayner et al. 2025; Shealy et al. 2025; Stubbs et al. 2017; Valdivieso et al. 2023), however the extent and direction of these patterns vary widely. In some cases, inclusion of sex even improves epigenetic clock performance. To investigate whether *S. tigrinum*, a species possessing a male heterogametic (XX/XY) sex determination system (Lee et al. 2025; Yamaguchi et al. 2022), exhibits sex‐associated DNAm variation, we performed differential methylation analysis between females (*n* = 30) and males (*n* = 21) across all ages using logistic regression with correction for overdispersion in the methylKit package as described previously. For the purposes of comparison to differentially methylated CpGs (DMCs) associated with age which were only identified in known‐age AB individuals, this analysis was also limited to AB individuals. Loci with an FDR < 0.05 were considered differentially methylated. Sex‐associated DMCs were annotated with respect to gene context, CpG island context, and distance to the closest TSS. Fisher's exact tests were performed to investigate whether sex‐associated DMCs were over‐ or underrepresented in genomic regions of interest and GO overrepresentation analysis was performed using the gene identities of TSSs proximate to DMCs as described previously.

## 3 Results

### 3.1 Global Methylation Patterns Reflect a Subtle Decline in Overall Methylation With Age

A total of 14,807,085 CpG sites (33.9% of all CpGs in the genome) was covered by at least 5 reads in 80% (*n* = 56) of samples. Average percent methylation across all covered CpGs declined subtly but significantly with age according to a simple linear regression (𝛽 = −0.055, *p* < 0.001; *R* ^2^ = 0.165; Figure 1B). Unbiased clustering of samples via principal components analysis did not indicate the presence of any aberrant samples (Figure 1C). The first principal component explained only 2.48% of the variation in methylation across samples but did exhibit an apparent asymptotic relationship with age. In accordance with this observation, PC1 exhibited a strong Spearman correlation with age (𝜌 = −0.76, *p* < 0.0001; Figure 1D).

### 3.2 Functionally Distinct CpG Sites Exhibit Age‐Associated Hyper‐ and Hypo‐Methylation

**FIGURE 2:**
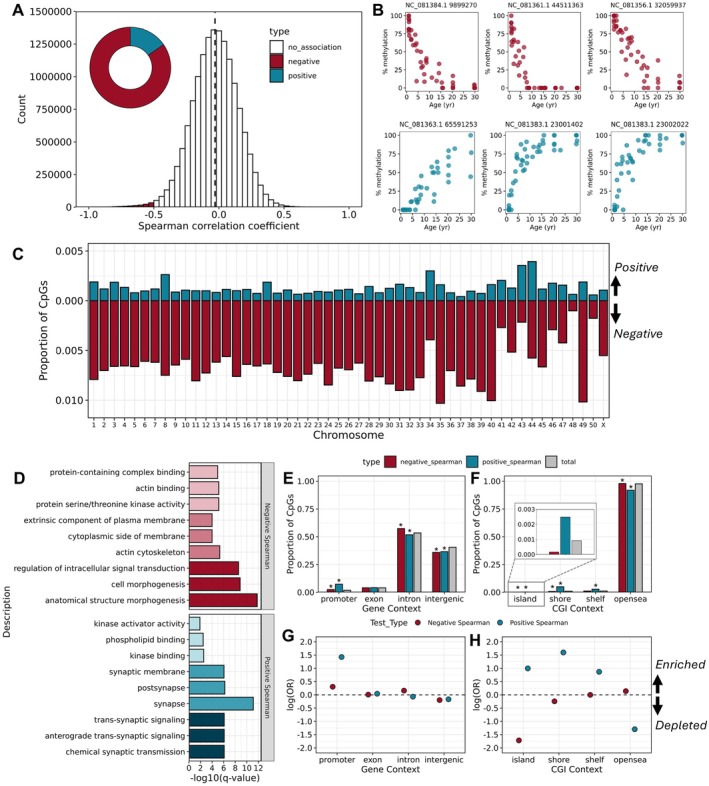
Genome‐wide age‐associated DNA methylation patterns. (A) Histogram depicting distribution of Spearman correlation coefficients with age for all filtered CpGs. Vertical dashed line indicates global mean correlation. Inset donut plot depicts proportion of CpGs exhibiting significant positive correlations (hypermethylation) with age (in blue) versus negative correlations (hypomethylation) with age (red) according to FDR < 0.05 and absolute correlation coefficient > 0.5. (B) Percent methylation across ages for loci exhibiting the top three strongest positive and negative age correlations. (C) Proportion of filtered CpGs per chromosome that are significantly correlated with age (positive correlations in blue, negative correlations in red). (D) Top three enriched gene ontology (GO) terms in each category (biological process, cellular component, molecular function) for genes in proximity to loci exhibiting negative or positive correlations with age. Colour saturation corresponds to different GO categories—darkest = biological process, middle = cellular component, lightest = molecular function. (E, F) Proportion of negatively and positively age‐associated CpGs overlapping different gene contexts and CpG island contexts. Grey bars depict proportion of all filtered CpGs that overlap with a given feature. Asterisks indicate a significant enrichment/depletion based on the results of a Fisher's exact test according to *p* < 0.05. (G, H) Log odds ratio of overlap with different gene or CpG island contexts. Values above zero indicate enrichment and values below zero indicate depletion.

A total of 158,015 CpGs (1.07% of filtered CpGs) exhibited a significant Spearman correlation with age and 112,532 CpGs (0.76% filtered CpGs) exhibited absolute Spearman correlation coefficients > 0.5 (Figure 2A,B). Many more loci exhibited negative correlations with age (i.e., greater methylation in younger individuals; *n* = 95,736; 85.1%) compared to positive correlations with age (i.e., greater methylation in older individuals; *n* = 16,796; 14.9%; Figure 2A inset). Further, the overall mean Spearman correlation coefficient across all filtered CpG sites was −0.032 indicating widespread loss of methylation with age, a pattern consistent with the decline in global average percent methylation with age (Figure 1B). Similar patterns were observed when considering Pearson correlations. A total of 95,232 CpGs exhibited a significant Pearson correlation with age and 77,708 CpGs exhibited absolute Pearson correlation coefficients > 0.5. Of those, 67,053 sites (86.3%) were negatively correlated with age and 10,655 sites (13.7%) were positively correlated with age. We describe results for loci exhibiting age‐associated methylation changes according to Spearman correlations moving forward, however, these are consistent with our results when only considering Pearson correlations (Figure S2).

Positively and negatively age‐associated loci were distributed relatively evenly across chromosomes (Figure 2C), however, these sites differed markedly in their distribution with respect to genes and CpG islands. Positively age‐associated loci were strongly enriched in promoter regions (odds ratio = 4.17; *p* < 0.0001) and depleted in intron (odds ratio = 0.93; *p* < 0.0001) and intergenic regions (odds ratio = 0.85; *p* < 0.0001; Figure 2E,G). Similarly, negatively age‐associated loci were enriched in promoters but to a lesser degree (odds ratio = 1.36; *p* < 0.0001) and depleted in intergenic regions (odds ratio = 0.82; *p* < 0.0001; Figure 2E,G). By contrast, negatively age‐associated loci were enriched in introns (odds ratio = 1.17; *p* < 0.0001; Figure 2E,G). With respect to CpG islands, positively age‐associated loci were enriched in CpG‐dense islands (odds ratio = 2.71; *p* < 0.0001), shores (odds ratio = 4.95; *p* < 0.0001), and shelves (odds ratio = 2.39; *p* < 0.0001), and were depleted in CpG‐poor open sea regions (odds ratio = 0.27; *p* < 0.0001; Figure 2F,H). Negatively age‐associated loci exhibited a nearly opposite pattern wherein these sites were depleted in CpG islands (odds ratio = 0.18; *p* < 0.0001) and shores (odds ratio = 0.78; *p* < 0.0001) and were enriched in open sea regions (odds ratio = 1.15; *p* < 0.0001; Figure 2F,H). The genes in proximity to positively and negatively age‐associated loci exhibited distinct gene ontology enrichment and TF regulation (Figure 2D). The top enriched terms for genes associated with positive age‐correlations related to neuronal function and extracellular signalling and included “trans‐synaptic signalling”, “phospholipid binding”, and “kinase activator activity.” By contrast, the top enriched terms for genes associated with negative age‐correlations related to developmental processes and intracellular signalling and included “cell morphogenesis” and “cytoplasmic side of the membrane”. Further, genes with TSSs within 5 kb of positively age‐associated loci were enriched for regulation by RING1B (odds ratio = 2.21; *q* < 0.0001), SMAD4 (odds ratio = 2.01; *q* < 0.0001), ZNF217 (odds ratio = 2.27; *q* < 0.0001), and components of the Polycomb Repressive Complex 2—SUZ12 (odds ratio = 1.83; *q* < 0.0001), EZH2 (odds ratio = 2.13; *q* < 0.0001), and JARID2 (odds ratio = 2.41; *q* < 0.0001), among others (Table S2). Genes in proximity to negatively age‐associated loci were enriched for regulation by ZNF217 (odds ratio = 1.98; *q* < 0.0001) as well, but also TFAP2C (odds ratio = 1.94; *q* < 0.0001), HAND2 (odds ratio = 1.50; *q* < 0.0001), EP300 (odds ratio = 1.76; *q* < 0.0001), AR (odds ratio = 1.50; *q* < 0.0001), and NR3C1 (odds ratio = 1.46; *q* < 0.0001; Table S2).

**FIGURE 3:**
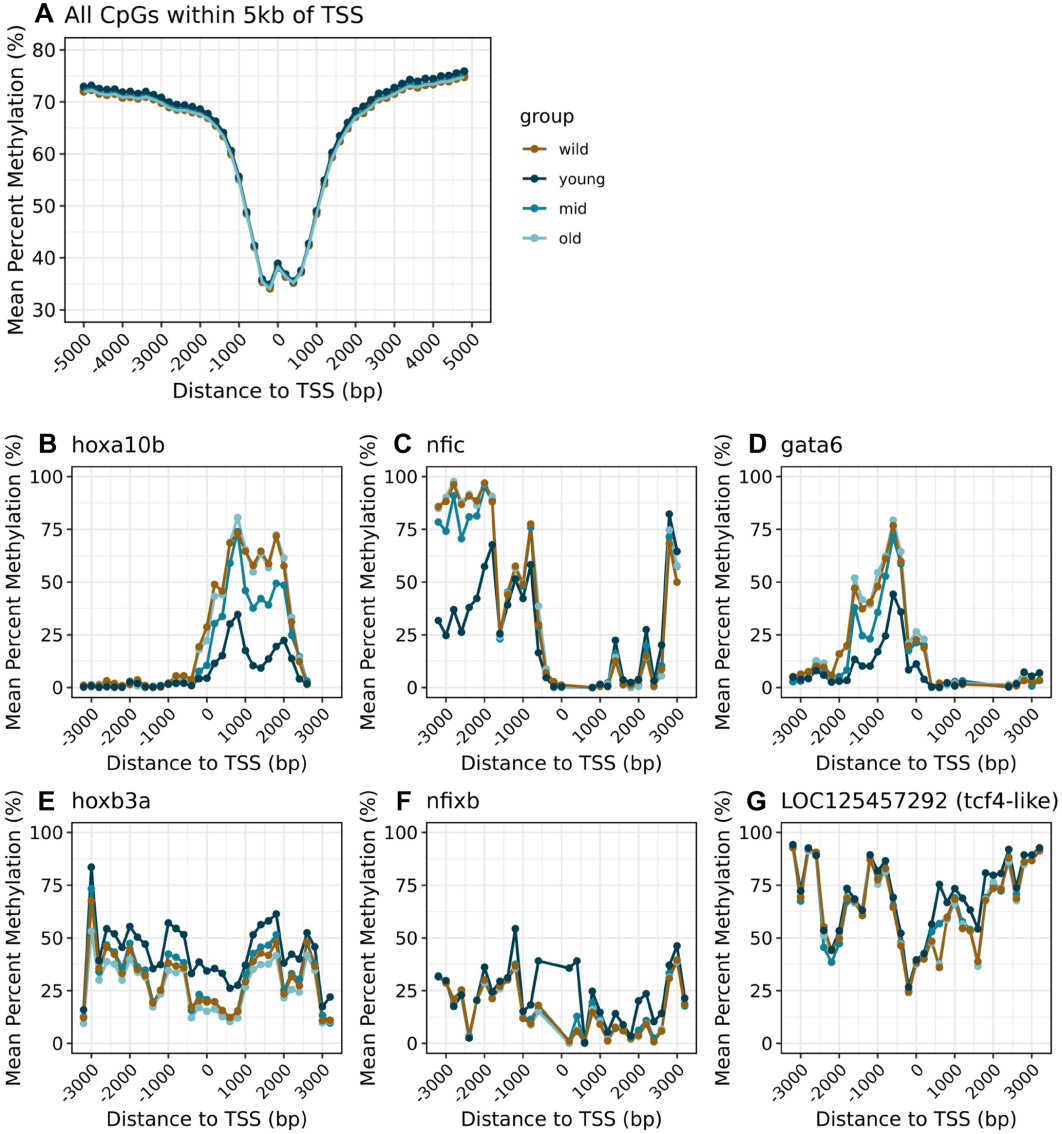
Age‐associated DNAm patterns near transcriptional start sites (TSSs). Plots depict patterns of mean percent methylation for 200 bp windows within 5 kb of a TSS grouped by categorical age group (A) genome‐wide, (B–D) for genes with the highest number of positively age‐associated CpGs within 5 kb of their TSS, and (E–G) for genes with the highest number of negatively age‐associated CpGs within 5 kb of their TSS. Brown = wild‐caught; dark blue = young (≤ 6.2 years); medium blue = mid (6.2 years < age ≤ 14.2 years), and light blue = old (> 14.2 years).

At a genome‐wide scale, methylation levels declined sharply within 2 kb upstream and downstream of transcriptional start sites (TSSs; Figure 3A), a pattern consistent with observations in other vertebrates (Feng et al. 2010). However, several genes with relevance to development and morphogenesis exhibited a clustering of age‐associated CpGs in proximity to their TSS. The three genes with the highest number of positively age‐associated sites within 5 kb of their TSS were *hoxa10b* (*n* = 85 age‐associated CpGs), *nfic* (*n* = 62 age‐associated CpGs), and *gata6* (*n* = 58 age‐associated CpGs; Figure 3B–D). The three genes with the highest number of negatively age‐associated sites within 5 kb of their TSS were *hoxb3a* (*n* = 245 age‐associated CpGs), *nfixb* (*n* = 80 age‐associated CpGs), and an uncharacterized gene predicted to be related to *tcf‐4* (*n* = 66 age‐associated CpGs; Figure 3E–G).

### 3.3 Age‐Associated Changes in Methylation Tend to Be More Pronounced Early in Life and Often Exhibit Contrasting Changes Later in Life

**FIGURE 4:**
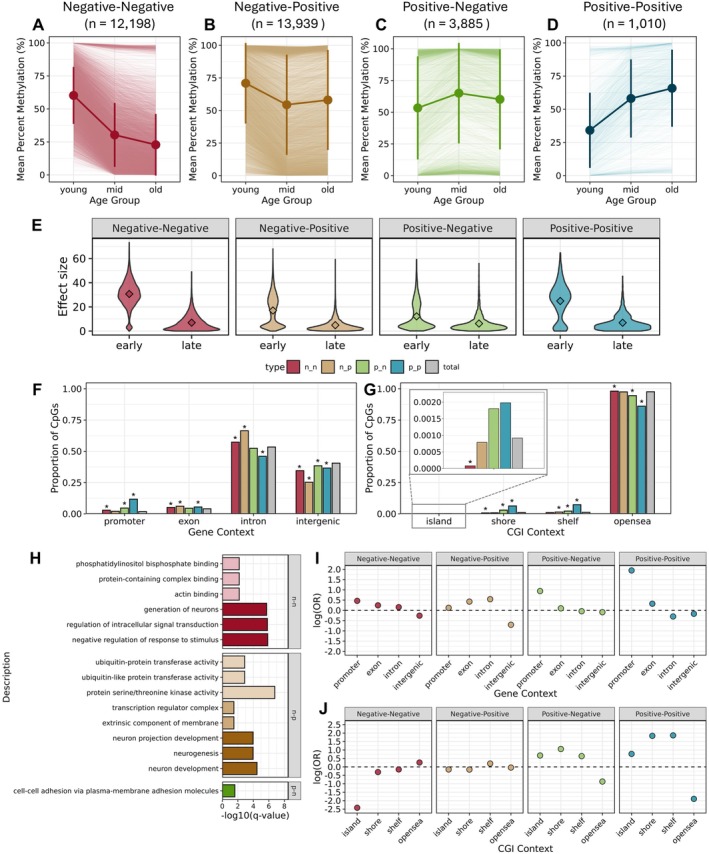
Patterns of age‐associated differential methylation across categorical age groups. Mean percent methylation across age groups for loci exhibiting different patterns of age‐association–(A) negative–negative, (B) negative–positive, (C) positive–negative, and (D) positive–positive. Central point ranges depict global group means and +/− 1 SD of the mean. (E) Absolute effect size of age group methylation differences early versus late in life. (F, G) Proportion of DMCs overlapping different gene contexts and CpG island contexts. Grey bars depict proportion of all filtered CpGs that overlap with a given feature. Asterisks indicate a significant enrichment or depletion based on the results of a Fisher's exact test according to *p* < 0.05. (H) Top three enriched gene ontology (GO) terms in each category (biological process, cellular component, molecular function) for genes in proximity to age‐associated DMCs. Colour saturation corresponds to different GO categories—darkest = biological process, middle = cellular component, lightest = molecular function. (I, J) Log odds ratio of overlap with different gene or CpG island contexts. Values above zero indicate enrichment and values below zero indicate depletion.

We identified DMCs between categorical age groups to characterize different age‐associated methylation trajectories. A total of 31,032 unique CpG sites (0.22% variance filtered CpGs) were significantly differentially methylated in at least one of the age‐group comparisons (Figure 4). The most common pattern of methylation change was one in which methylation declined with age early in life then subsequently increased with age later in life (“negative–positive”; *n* = 13,939, 44.9%; Figure 4B). The second most common pattern of methylation change was a consistent decline in methylation with age over the course of the lifespan (“negative–negative”; *n* = 12,198, 39.3%; Figure 4A). Fewer sites increased in methylation with age early in life before declining with age later in life (“positive–negative”; *n* = 3885, 12.5%; Figure 4C) and the least common pattern of methylation change was a consistent increase in methylation with age over the course of the lifespan (“positive–positive”; *n* = 1010, 3.2%; Figure 4D). Notably, across all four trajectories, age‐associated methylation changes tended to be more pronounced early in life (i.e., ‘young’ to ‘mid’ transition) than later in life (i.e., ‘mid’ to ‘old’ transition; Figure 4E). The subset of loci exhibiting consistent increases in methylation over the lifespan exhibited a strong enrichment in promoter regions (odds ratio = 7.00; *p* < 0.0001), CpG shores (odds ratio = 6.28; *p* < 0.0001), and shelves (odds ratio = 6.46; *p* < 0.0001), and strong underrepresentation in open sea regions (odds ratio = 0.15; *p* < 0.0001; Figure 4F,G,I,J). Loci exhibiting an increase in methylation early in life and loss of methylation later in life (“positive–negative”) similarly were enriched in promoters (odds ratio = 2.56; *p* < 0.0001), CpG shores (odds ratio = 2.88; *p* < 0.0001), and shelves (odds ratio = 1.89; *p* < 0.0001) and were underrepresented in open sea regions (odds ratio = 0.42; *p* < 0.0001; Figure 4F,G,I,J).

Loci exhibiting a loss of methylation early in life and gain in methylation late in life (“negative–positive”) exhibited a distinct genomic distribution compared to loci exhibiting a consistent loss of methylation with age (“negative–negative”; Figure 4F,G,I,J). For example, sites that consistently lost methylation were strongly depleted in CpG islands (odds ratio = 0.74; *p* = 0.002) and enriched in open seas (odds ratio = 1.30; *p* < 0.0001), while sites exhibiting an early loss of methylation followed by an increase in methylation did not significantly differ from background in their overlap with CpG islands or open seas, and were enriched in shelves (odds ratio = 1.22; *p* = 0.006). These subsets of age‐associated sites co‐localized with genes of different functional categories. Genes in proximity to sites consistently losing methylation with age were enriched for GO terms including “actin binding”, “phosphatidylinositol biphosphate binding”, “negative regulation of response to stimulus”, and “regulation of intracellular signal transduction”. By contrast, genes proximal to sites that initially lost methylation then gained methylation with age were enriched for GO terms including “protein serine/threonine kinase activity”, “ubiquitin‐protein transferase activity”, and “neuron development” (Figure 4H).

### 3.4 Epigenetic Clocks Predict Age With High Accuracy

**FIGURE 5:**
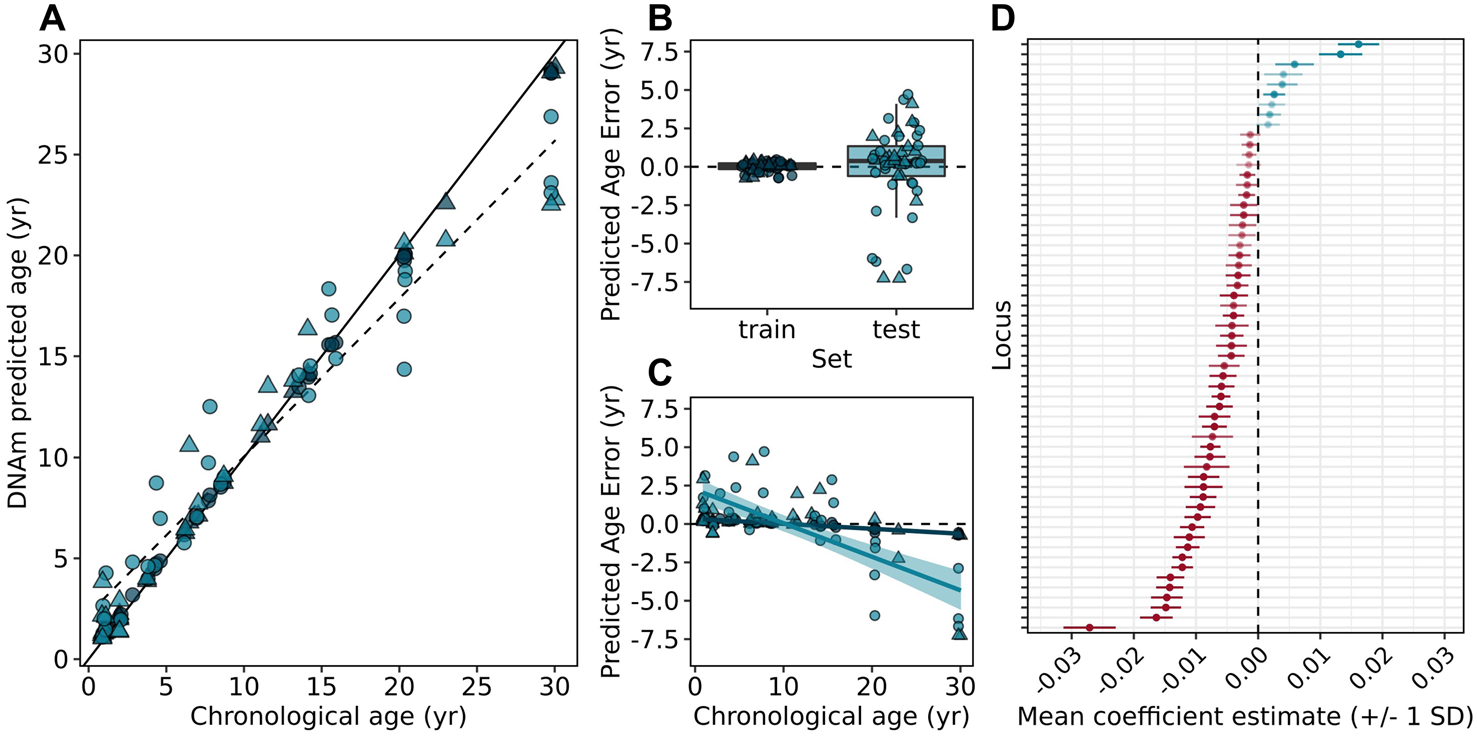
Calibration and performance of epigenetic age estimator via leave‐one‐out cross validation. (A) Age predictions based on cross‐validated elastic net regression by known chronological age. Training set age predictions (dark blue) are mean ages predicted across all models that included the training sample. Test set age predictions (light blue) indicate the predicted age when the sample was left out of model fitting. Solid line indicates 1:1 line. Dashed line indicates linear regression line for test set predictions. Values are indicated by circles for females and triangles for males. (B) Boxplot depicting difference between predicted age and known chronological age for the training and test sets. (C) Relationship between predicted age error and chronological age. Light blue regression line depicts tendency to underestimate ages of older test samples. (D) Coefficient estimates (mean +/− 1SD) for CpGs selected to be included in at least 40 of 51 models (*n* = 59). Transparency depicts the proportion of models in which a given CpG was selected to be included in—less transparent corresponds to inclusion in a higher proportion of models. Red = negative coefficients, Blue = positive coefficients.

Across all calibration approaches, the DNAm status of less than 100 CpGs predicted age in zebra sharks with high accuracy (e.g., within 1–4 years; Figure 5 and Table S3). In the leave‐one‐out cross‐validation (LOOCV) approach, between 56 and 90 CpGs were selected to be included in the predictive model of age, 23 of which were selected to be included across all models and 59 of which were selected to be included in at least 40 of 51 models (Figure 5D and Table S4). Of those sites included in at least 40 of 51 models, 9 (~15.3%) had positive model coefficients and 50 (~84.7%) had negative model coefficients. The predicted DNAm age and chronological age of test samples were highly correlated (*r* = 0.97) with a median absolute error (MAE) of 1.03 years (~3.4% of maximum age). However, model performance tended to decline in the oldest individuals (Figure 5C). The MAE of the test set was 0.72 years for individuals under 20 years old and 3.32 years for individuals over 20 years old. Limiting potential predictors to age‐associated sites (absolute *r* > 0.5) in the training set did not improve model performance (Table S3). Assigning higher observation weights to mature individuals relative to immature individuals marginally improved age predictions for individuals over 20 years old (weighted test MAE = 3.16; unweighted test MAE = 3.32) but compromised overall model performance (overall test MAE = 1.21). When a single training set of AB individuals (*n* = 41) was used to calibrate a predictive model of age, 70 CpGs were selected as predictors (Table S5) and the resulting model predicted age in the naïve test set (*n* = 11) with a MAE of 1.31 years (~4.3% of maximum age; *r* = 0.98; Table S3 and Figure S3).

When a simple linear model was fit to predict age based on the top 10 or top 20 most age‐associated sites (according to Pearson's *r*), the resulting model still performed relatively well (Figure S4). In the LOOCV approach, the models based on the top 10 age‐associated sites predicted ages in the test set with an MAE of 1.62 years (~5.4% of maximum age; *r* = 0.90; Table S3 and Figure S4). The models based on the top 20 age‐associated sites predicted ages in the test set with an MAE of 1.99 years (~6.6% of maximum age; *r* = 0.92; Table S3).

**FIGURE 6:**
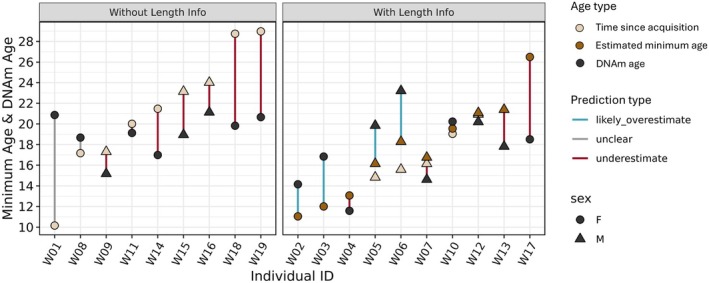
Performance of epigenetic age estimator on wild‐caught individuals within unknown chronological ages. Light brown points indicate time since acquisition, dark brown points indicate size‐corrected minimum age, and black points indicated DNAm predicted age based on an elastic net regularized regression model trained on all aquarium‐bred animals. Blue lines indicate likely but uncertain overestimates. Red lines indicate age underestimates. Grey lines indicate errors between minimum and predicted age that are of unknown cause (e.g., prediction error versus true difference between chronological and minimum age).

Assessing performance of the age predictor on wild‐caught (WC) individuals of unknown chronological age presents technical challenges stemming from the inability to clearly distinguish sources of prediction error. However, 10 of the 19 WC individuals included in this study were measured following aquarium acquisition to obtain data on total length. We used this data to generate size‐corrected minimum age estimates based on a von Bertalanffy growth function (Figure S5). For the remaining 9 WC individuals, time since aquarium acquisition was treated as minimum age. When data from all 51 AB individuals were used to calibrate an age predictor using elastic net regularized regression, 89 CpG sites were selected as predictors (Table S6). For WC individuals with total length information, the MAE between predicted age and size‐corrected minimum age was 3.34 years (~11.1% of maximum age; Table S3). In half of cases (*n* = 5), DNAm predicted age underestimated size‐corrected minimum age and, in turn, chronological age (Figure 6). In the remaining cases, DNAm predicted age overestimated size‐corrected minimum age indicating a likely but uncertain overestimation of chronological age (Figure 6). For WC individuals without total length information, the MAE between predicted age and minimum age was 4.19 years (Figure 6). One individual's predicted age was over 10 years older than the corresponding minimum age. An additional blood sample from this individual was sequenced to rule out technical contributions to the error and the predicted age of the new sample confirmed the previous result (Appendix S1) indicating this individual was likely acquired by an aquarium as an adult. Excluding this individual, the MAE between predicted age and minimum age was 3.54 years. However, predicted age underestimated minimum age in seven of eight cases suggesting the true prediction error is greater than this estimate (Figure 6). Across all calibration approaches, models did not vary in their predictive performance between females and males (e.g., LOOCV—Mann–Whitney *U*‐test: *W* = 296, *p* = 0.73). Further, calibration of sex‐specific age predictors did not improve model performance.

### 3.5 Sex‐Associated DNAm Variation Is More Limited Than Age‐Associated Variation

Many fewer DMCs were associated with sex than were associated with age. A total of 476 CpG sites exhibited sex‐associated differential methylation, the majority (68.5%) of which exhibited greater methylation in females (Figure S6). Only two sex‐biased DMCs were located on the X chromosome; however, due to the low number of filtered CpGs on the X, this resulted in the X chromosome having the highest proportion of filtered CpGs exhibiting sex‐biased differential methylation of any chromosome. Further, the filtered CpGs located on the X chromosome did not exhibit female‐biased hypermethylation on a broad scale (Figure S6). Sex‐associated DMCs exhibiting female‐biased methylation were enriched in exons (odds ratio = 2.93; *p* < 0.0001) and introns (odds ratio = 2.18; *p* < 0.0001) and depleted in intergenic regions (odds ratio = 0.29; *p* < 0.0001). By contrast, DMCs exhibiting male‐biased methylation were enriched in promoter regions (odds ratio = 4.15; *p* = 0.00014). Male‐biased DMCs overlapped the promoters of nine unique genes including *nrarpa*, *slc7a2*, *nhlh2*, *klf5l*, *tmem135*, and *dpep2*. In addition, a cluster of female‐biased DMCs of high effect size (mean sex difference = 40.1%) clustered within an exon and downstream of a gene predicted to be *zinc finger protein 235*.

## 4 Discussion

In the present study, we demonstrate that DNAm patterns at a subset of loci in blood samples predict chronological age with high accuracy in the zebra shark. Loci included in epigenetic clocks for this species represent only a small proportion of the age‐associated DNAm patterns occurring across the genome. In fact, the first major axis of DNAm variation among samples according to principal components analysis showed a strong asymptotic relationship with age, indicating that age is a prominent driver of epigenome‐wide patterns in zebra shark blood cells, especially in early life. While the functional consequences of these age‐associated methylation patterns are unknown, their genomic distribution highlights key similarities to epigenomic aging dynamics in other vertebrates.

Genome‐wide loss of methylation with increasing age was one of the earliest reported age‐associated epigenetic alterations in humans (Bollati et al. 2009; Fuke et al. 2004; Heyn et al. 2012) and has been observed in several non‐mammalian vertebrates (Nilsen et al. 2016; Parrott et al. 2014). Consistent with these findings, we observed global mean methylation across all CpGs in our dataset decline slightly with age. Further, the number of individual sites for which methylation level is significantly and negatively correlated with age outnumbered those that are positively correlated with age. Such widespread age‐associated hypomethylation is thought to reflect a largely passive process resulting from decreased fidelity of epigenetic maintenance mechanisms over the course of cell divisions affecting regions of the genome that tend to be highly methylated (e.g., repetitive elements, gene bodies; Bollati et al. 2009; Endicott et al. 2022; Huh et al. 2013; Jones 2012). Indeed, in the present study, loci exhibiting age‐associated hypomethylation were enriched in introns and regions of low CpG density and depleted in CpG islands. Loci losing methylation with age also tended to co‐localize with genes regulated by zinc finger protein 217 (ZNF217) and transcription factor AP‐2 gamma (TFAP2C), two transcription factors associated with human tumour progression (Banck et al. 2009; Orso et al. 2008).

Loci that gained methylation with age occurred in regions of the zebra shark genome largely distinct from those that lost methylation with age. Mirroring patterns observed in mammals (Lu, Fei, et al. 2023), loci that gained methylation with age were enriched in regions of high CpG density including CpG islands, shores, and shelves, were depleted in regions of low CpG density, and co‐localized with gene targets of Polycomb repressive complexes (PRC1 subunit RING1B; PRC2 subunits SUZ12, EZH2, and JARID2). These findings are consistent with the hypothesis that age‐associated gains and losses of methylation are driven by functionally distinct processes (Moqri et al. 2025). Interestingly, age‐associated hypermethylation in regions regulated by PRC2 tends to be highly conserved across mammal species and tissue types (Lu, Fei, et al. 2023; Moqri et al. 2024; Wilkinson et al. 2021) and may represent a nexus point causally linking development and aging processes at the epigenomic level (Aldiri and Vetter 2012; Lu, Tian, and Sinclair 2023). Taken together, our results in the zebra shark extend these observations outside of mammals and suggest regions gaining methylation with age in association with PRC2 target genes warrant prioritization as potential hotspots of conserved epigenetic aging signals across vertebrates.

The methylome does not change uniformly with age over the course of the zebra shark lifespan. When comparing categorical age groups, methylation differences between young and middle‐aged individuals tended to be greater in magnitude than those between middle‐aged and old individuals. This result is congruent with past observations in both mammals and some non‐mammals that age‐related epigenetic changes are more dynamic early in life (Bertucci et al. 2021; Horvath 2013; Shealy et al. 2025; Wang et al. 2020), a pattern hypothesized to result, in part, from increased growth and developmental progression in juveniles (Horvath and Raj 2018; Raj and Horvath 2020). It should be noted that a considerable proportion of our samples (21 of 51) were derived from individuals that had not reached sexual maturity. Whether epigenetic changes occurring in early life represent “aging” per se is the subject of active debate (Gladyshev et al. 2024). Even so, it is interesting to consider how early epigenetic changes may shape later‐life epigenetic aging trajectories. Accelerated epigenetic aging in early life has been linked to juvenile growth in endotherms (Haller et al. 2025; Simpkin et al. 2017). For example, great tits ( *Parus major* ) reared in conditions with higher resource availability as a result of reduced conspecific competition showed accelerated epigenetic age relative to individuals reared in control broods (Haller et al. 2025). Given that growth and development in ectotherms are sensitive to ambient environmental conditions, it is worth considering how potential links between rising ocean temperatures and early life growth patterns could influence epigenetic aging trajectories in wild populations. Investigations into the relationships between growth, organismal aging phenotypes, and environmental factors like temperature in ectotherms have focused primarily on telomere length and DNA repair mechanisms (Bae et al. 2021; Bronikowski 2008; Dupoué et al. 2022; Friesen et al. 2022; Hansson et al. 2024; Schwartz and Bronikowski 2013). Thus, expansion of epigenetic aging datasets in ectotherms, like the one presented here, will open new opportunities to uncover the mechanisms by which environmental factors shape life history tradeoffs.

Beyond differences in the magnitude of epigenetic change observed throughout the zebra shark lifespan, over half of age‐associated differentially methylated sites exhibited contrasting directions of methylation change early in life versus late in life. Such non‐linear, non‐monotonic patterns are a common feature of aging methylomes (Okada et al. 2023; Olecka et al. 2024), yet are understudied across all taxa. For example, it is unclear if non‐monotonic changes to DNAm result from the same underlying processes that drive consistent, linear patterns, nor how alterations to the mode and tempo of these changes relate to organismal phenotypes. These patterns also present challenges from a modelling perspective, especially in the context of generating age predictors. For instance, many epigenetic clocks, including the ones we present here, exhibit decreased performance in older animals (Lu, Fei, et al. 2023), due in part to the inclusion of loci with stronger patterns of age‐association early in life. The frequency of such non‐monotonic pattens of epigenetic aging combined with reliance on linear modelling approaches to construct age predictors compromises our understanding of punctuated and stage‐specific epigenetic changes over the life course (Olecka et al. 2025). Future studies of non‐linear epigenetic aging trajectories, especially those employing longitudinal sampling, are likely to yield insights into the drivers of these patterns and how they might be used to improve age prediction models.

The utility of the zebra shark epigenetic clocks reported here in conservation contexts will depend on their intended application. Epigenetic clocks can be tailored to balance trade‐offs between the number of constituent CpGs, accuracy, and identifying mismatches between chronological and biological age. If using clocks to estimate population age structures across large numbers of individuals, clocks relying on the fewest CpGs are more easily applied using approaches such as multiplex PCR of bisulfite treated DNA and subsequent amplicon sequencing (Piferrer and Anastasiadi 2023). In the present study, we calibrated epigenetic clocks according to two primary aims: (1) To predict ages of zebra sharks across the entire lifespan with high accuracy, and (2) To generate sparse models that rely on methylation information from relatively few loci. Using as a few as 10 loci, we show accuracy is minimally sacrificed. At the same time, due to their sparsity and reliance on age‐associated methylation loss, the clocks reported here have reduced performance in the oldest animals and provide limited information about biological age acceleration. In the same vein, it is unclear whether these clocks would extend directly to other tissues or species. To achieve these aims, adjusted clock calibration approaches will need to be implemented that incorporate additional phenotypic information (e.g., metrics of physiological health), expand the scope of training samples, or prioritize different model features (e.g., locus conservation across species).

Epigenetic clocks derived from blood samples represent a non‐lethal and relatively non‐invasive approach for estimating individual age, features that are critical for use in conservation and management. Like a large proportion of other elasmobranchs, the zebra shark is currently listed as Endangered by the IUCN Red List of Threatened Species (Rigby et al. 2024). Consequently, epigenetic clocks in this species provide unique opportunities to inform population assessments but also present a particular challenge due to the difficulty of obtaining calibration samples. Current approaches to constructing epigenetic clocks rely on an initial cohort of known‐aged individuals (Mayne et al. 2021), and here, we trained our clocks on known‐aged samples from individuals born and maintained in aquariums. However, the extent to which our reliance on samples from aquarium‐bred individuals influences age estimates of wild, free‐living individuals is difficult to determine. This point is highlighted in our analyses of wild‐caught individuals in which sources of error in DNAm‐based age estimates cannot be fully parsed between model error, true variation in epigenetic age, and differences between time since acquisition and chronological age. The ramifications of using aquarium‐ or zoo‐reared cohorts to construct clocks in other species will likely vary depending on the species and the extent to which conditions (e.g., diet, stress, etc.) of training set individuals approximate the physiology of wild individuals. Results presented here underscore the critical role aquarium and zoo specimens can serve as known‐age calibration populations and raise considerations for their use in future efforts to develop DNAm‐based age estimators in threatened species. As more epigenetic clocks are developed, comparisons between free‐living individuals and individuals reared in zoos or aquariums will inform our understanding of sources of variation in epigenetic aging and advance efforts to maximize the utility of epigenetic tools in conservation contexts. Taken together, this study presents a reliable epigenetic clock for the zebra shark and contributes a valuable genome‐wide view of age‐associated DNAm patterns in an elasmobranch thereby providing a basis for the identification of conserved epigenetic aging patterns in other species.

## Author Contributions

S.L.B., K.Ly., G.J.P.N, and B.B.P. conceptualized the study. K.Ly., J.W., L.A., N.K., A.J., E.L., T.H., J.A., D.P., A.F., J.O., K.A., C.S., K.Le., G.A.M., S.V., A.R., L.W., A.L., J.H., and M.S.contributed to sample collection. S.L.B. and L.Y. performed nucleic acid extraction and sequencing library preparation. S.L.B. performed data analysis. S.L.B. and B.B.P. wrote the original manuscript draft. B.B.P. and G.J.P.N. acquired funding for the study. A.B. acquired funding for support of S.L.B. All authors contributed to review and editing of the final manuscript.

## Funding

This work was supported by the National Science Foundation, 2232270, 2213824. U.S. Department of Energy, DE‐EM0004391.

## Disclosure

This report was prepared as an account of work sponsored by an agency of the United States Government. Neither the United States Government nor any agency thereof, nor any of their employees, makes any warranty, express or implied, or assumes any legal liability or responsibility for the accuracy, completeness or usefulness of any information, apparatus, product, or process disclosed, or represents that its use would not infringe privately owned rights. Reference herein to any specific commercial product, process, or service by trade name, trademark, manufacturer, or otherwise does not necessarily constitute or imply its endorsement, recommendation, or favouring by the US Government or any agency thereof. The views and opinions of the authors expressed herein do not necessarily state or reflect those of the US Government or any agency thereof.

## Conflicts of Interest

The authors declare no conflicts of interest.

## Supporting information


**Table S1:** Sample information and sequencing summary statistics.
**Table S2:** Transcription factor regulation of genes proximate to age‐associated loci.
**Table S3:** Comparison of epigenetic age estimators.
**Table S4:** Epigenetic clock coefficient estimates from calibration with leave‐one‐out cross validation.
**Table S5:** Epigenetic clock coefficient estimates from calibration with single training and test set.
**Table S6:** Epigenetic clock coefficient estimates from calibration with aquarium‐bred individuals as training set and wild caught individuals as test set.

**Figure S1:** Bioinformatic pipeline and epigenetic clock calibration approach.

**Figure S2:** Genome‐wide age‐associated DNA methylation patterns according to Pearson correlations. (A) Histogram depicting distribution of Pearson correlation coefficients with age for all filtered CpGs. Vertical dashed line indicated global mean correlation. Inset donut plot depicts proportion of CpGs exhibiting significant positive correlations (hypermethylation) with age (in blue) versus negative correlations (hypomethylation) with age (red) according to FDR < 0.05 and absolute correlation coefficient > 0.5. (B) Percent methylation across ages for loci exhibiting the top three strongest positive and negative age correlations. (C) Proportion of filtered CpGs per chromosome that are significantly correlated with age (positive correlations in blue, negative correlations in red). (D) Top three enriched gene ontology (GO) terms in each category (biological process, cellular component, molecular function) for genes in proximity to loci exhibiting negative or positive correlations with age. Colour saturation corresponds to different GO categories—darkest = biological process, middle = cellular component, lightest = molecular function. (E, F) Proportion of negatively and positively age‐associated CpGs overlapping different gene contexts and CpG island contexts. Grey bars depict proportion of all filtered CpGs that overlap with a given feature. Asterisks indicate a significant enrichment/depletion based on the results of a Fisher's exact test according to *p* < 0.05. (G, H) Log odds ratio of overlap with different gene or CpG island contexts. Values above zero indicate enrichment and values below zero indicate depletion.

**Figure S3:** Calibration and performance of epigenetic age estimator with single training and test set. (A) Age predictions based on single elastic net regression. Dark blue = training set predictions, light blue = test set predictions. Solid line indicates 1:1 line. Dashed line indicates linear regression line for test set predictions. (B) Boxplot depicting difference between predicted age and known chronological age for the training and test sets. (C) Relationship between predicted age error and chronological age. Light blue regression line depicts tendency to underestimate ages of oldest test samples.

**Figure S4:** Performance of epigenetic age estimators based on top 10 age‐associated CpGs in training set based on Pearson correlations. (A–C) Results from linear model calibrated via leave‐one‐out approach (D–F) Results from linear model calibrated with a single training and test set. (G) Results from linear model trained on all aquarium‐bred samples and tested on wild‐caught samples.

**Figure S5:** Estimating age for wild‐caught zebra sharks. (A) Individual growth trajectories of 43 aquarium bred zebra sharks from 0 to 2555 days of age. (B) von Bertalanffy growth function fitted using non‐linear mixed effects model with random intercepts for individual ID. Ribbon around line indicates 95% prediction interval based on 10,000 simulations of fixed effect parameter sets according to the variance–covariance matrix of estimates. Brown squares indicate age predictions from total length for wild‐caught individuals calculated from inverse of von Bertalanffy growth function.

**Figure S6:** Genome‐wide sex‐associated DNA methylation patterns. (A) Proportion of filtered CpGs per chromosome that are significantly differentially methylated between females and males (CpGs with female‐biased methylation in pink, CpGs with male‐biased methylation in blue). Inset donut plot depicts proportion of CpGs exhibiting differential methylation with respect to sex that show female biased methylation versus male‐biased methylation. (B, C) Proportion of sex‐associated CpGs overlapping different gene contexts and CpG island contexts. Grey bars depict proportion of all filtered CpGs that overlap with a given feature. Asterisks indicate a significant enrichment/depletion based on the results of a Fisher's exact test according to *p* < 0.05. (D, E) Log odds ratio of overlap with different gene or CpG island contexts. Values above zero indicate enrichment and values below zero indicate depletion. (F) Distribution of methylation status for all filtered CpGs located on the X chromosome in females and males. Central point indicates global mean.

**Appendix S1:** mec70326‐sup‐0008‐AppendixS1.pdf.

## Data Availability

Raw sequencing reads for whole‐genome enzymatic methyl‐sequencing libraries are publicly available in the NCBI Sequence Read Archive (SRA) under the BioProject PRJNA1422842. Associated metadata and additional body size data used for growth curve construction are available in the Dryad Digital Repository (https://doi.org/10.5061/dryad.dr7sqvbbb) and scripts used in data analysis are available in a public GitHub repository (https://github.com/slbock/elasmo_dnam_aging).
