# Supplementary figures and images for: Genome‐Wide DNA Methylation Patterns Predict Age in the Zebra Shark (Stegostoma tigrinum) and Provide Insight Into the Evolution of Vertebrate Aging
Source: Mol Ecol. 2026 Apr 3;35(7):e70326. doi: 10.1111/mec.70326 (PMC13047888; doi:10.1111/mec.70326)

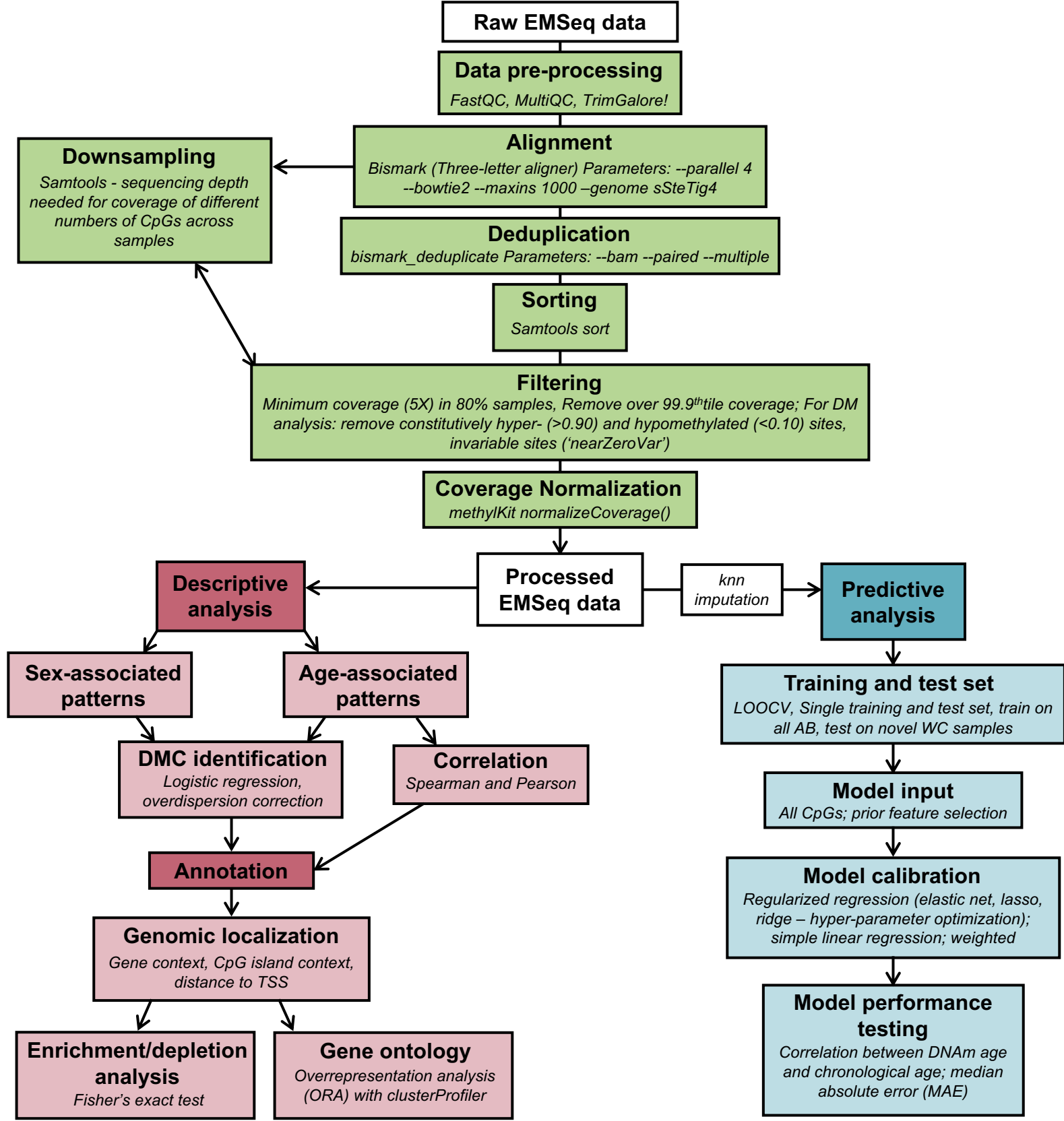

Supplement: Supplementary file 2 — Figure S1: Bioinformatic pipeline and epigenetic clock calibration approach. [file MEC-35-e70326-s001.pdf]

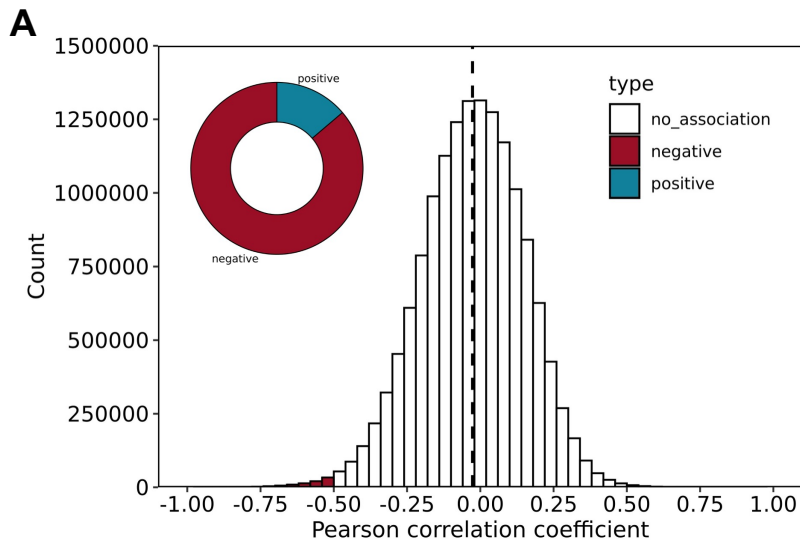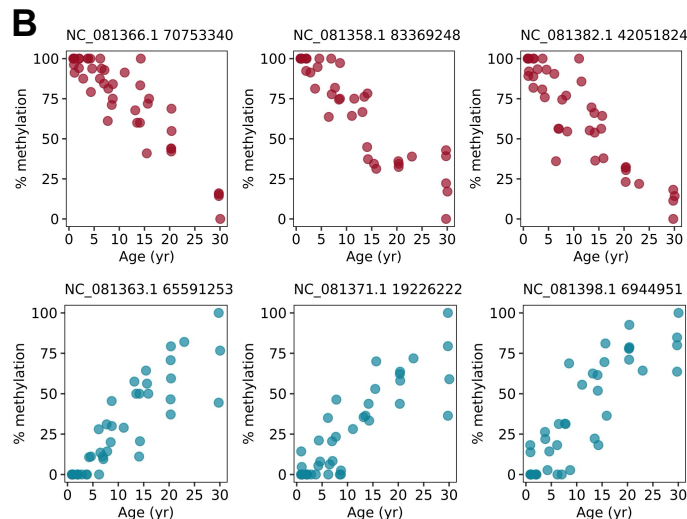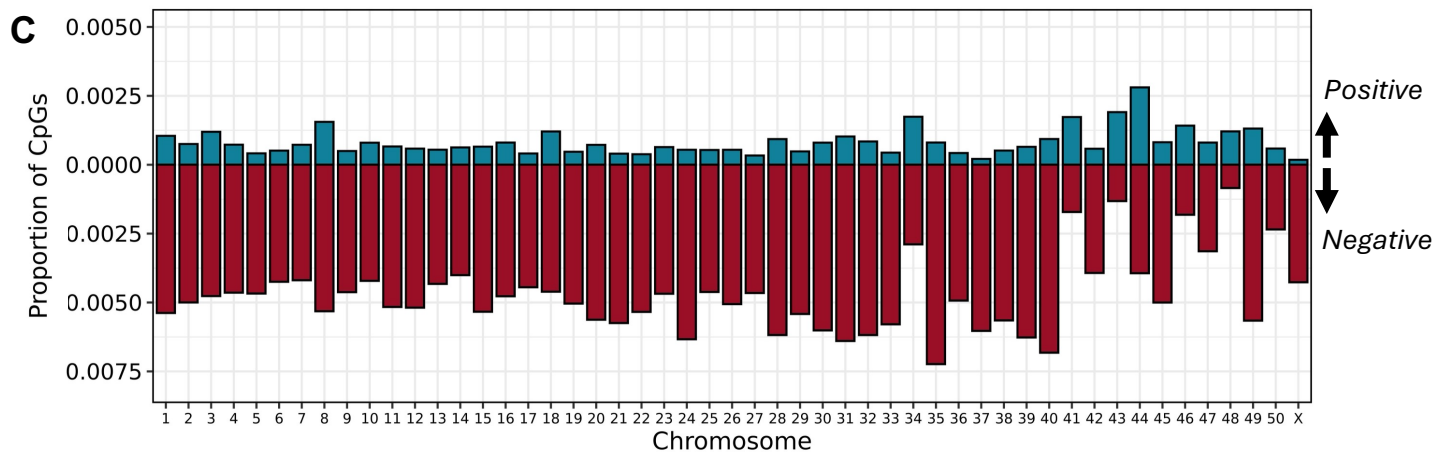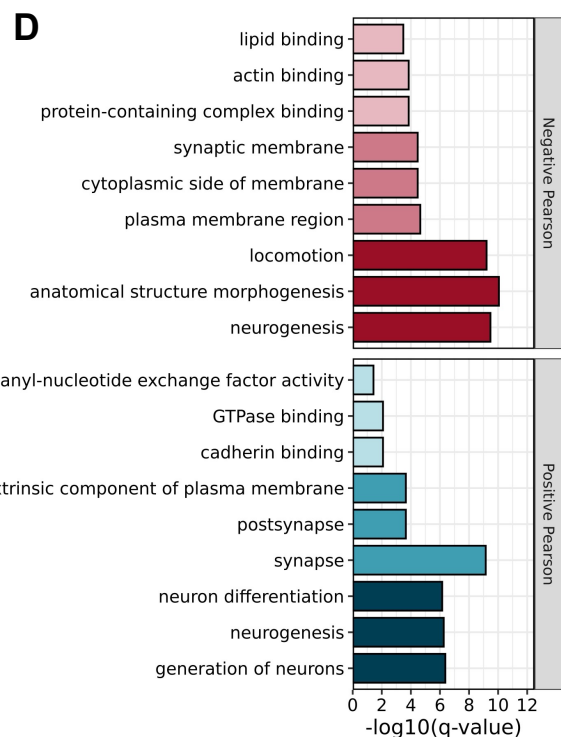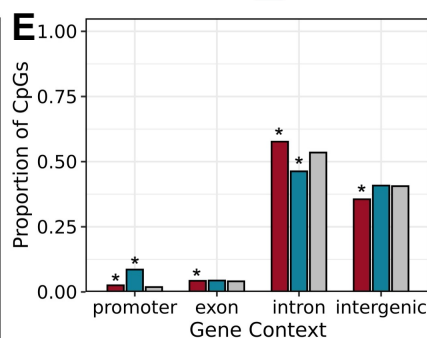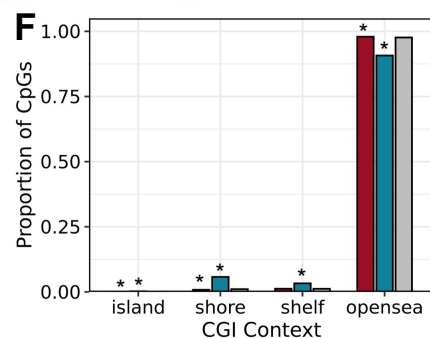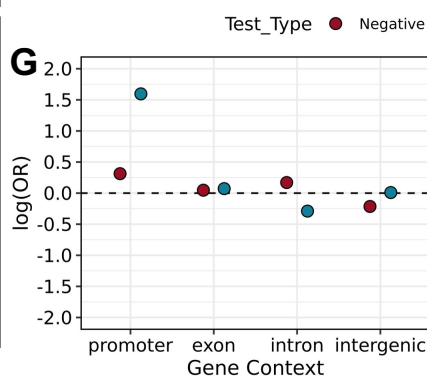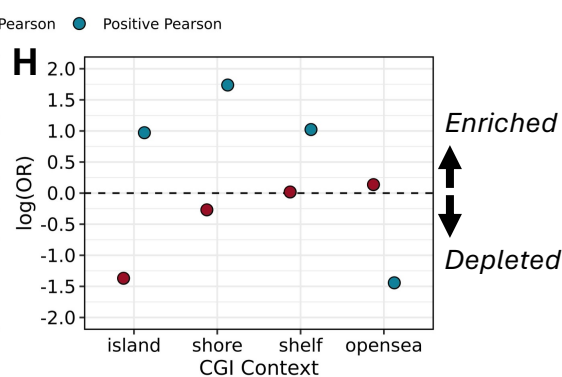

Supplement: Supplementary file 3 — Figure S2: Genome‐wide age‐associated DNA methylation patterns according to Pearson correlations. (A) Histogram depicting distribution of Pearson correlation coefficients with age for all filtered CpGs. Vertical dashed line indicated global mean correlation. Inset donut plot depicts proportion of CpGs exhibiting significant positive correlations (hypermethylation) with age (in blue) versus negative correlations (hypomethylation) with age (red) according to FDR < 0.05 and absolute correlation coefficient > 0.5. (B) Percent methylation across ages for loci exhibiting the top three strongest positive and negative age correlations. (C) Proportion of filtered CpGs per chromosome that are significantly correlated with age (positive correlations in blue, negative correlations in red). (D) Top three enriched gene ontology (GO) terms in each category (biological process, cellular component, molecular function) for genes in proximity to loci exhibiting negative or positive correlations with age. Colour saturation corresponds to different GO categories—darkest = biological process, middle = cellular component, lightest = molecular function. (E, F) Proportion of negatively and positively age‐associated CpGs overlapping different gene contexts and CpG island contexts. Grey bars depict proportion of all filtered CpGs that overlap with a given feature. Asterisks indicate a significant enrichment/depletion based on the results of a Fisher's exact test according to p < 0.05. (G, H) Log odds ratio of overlap with different gene or CpG island contexts. Values above zero indicate enrichment and values below zero indicate depletion. [file MEC-35-e70326-s006.pdf]

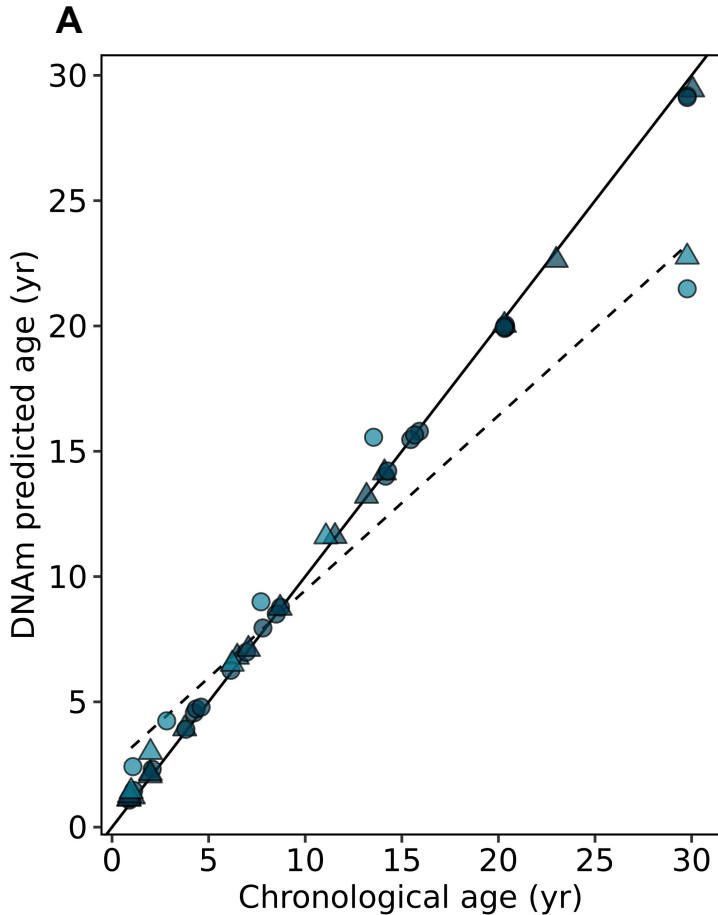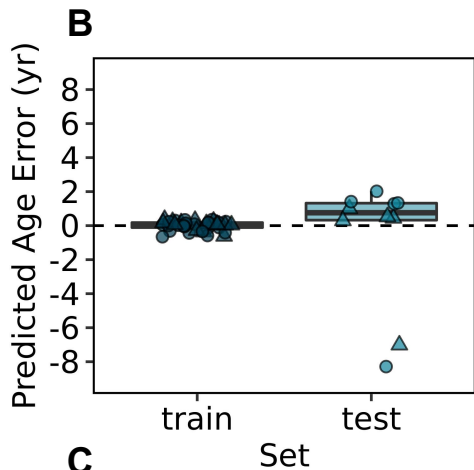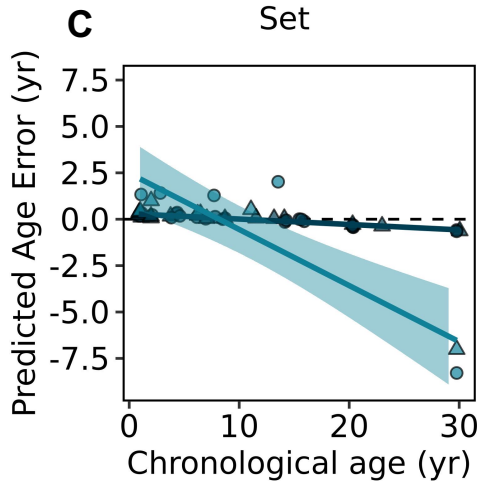

Supplement: Supplementary file 4 — Figure S3: Calibration and performance of epigenetic age estimator with single training and test set. (A) Age predictions based on single elastic net regression. Dark blue = training set predictions, light blue = test set predictions. Solid line indicates 1:1 line. Dashed line indicates linear regression line for test set predictions. (B) Boxplot depicting difference between predicted age and known chronological age for the training and test sets. (C) Relationship between predicted age error and chronological age. Light blue regression line depicts tendency to underestimate ages of oldest test samples. [file MEC-35-e70326-s004.pdf]

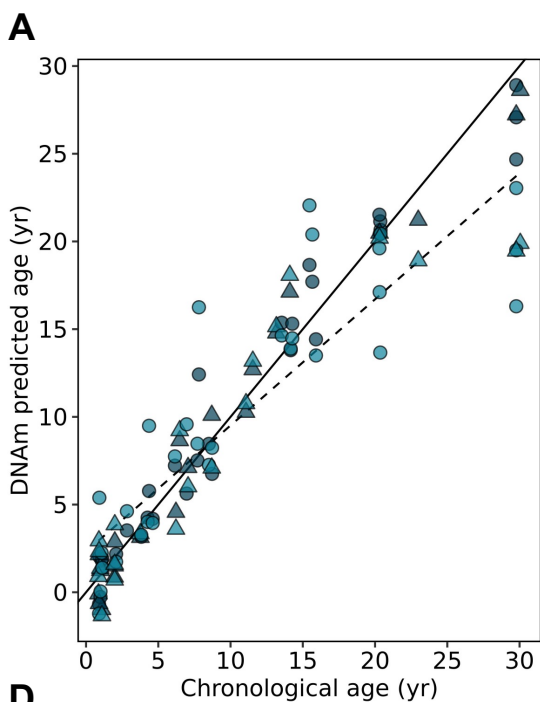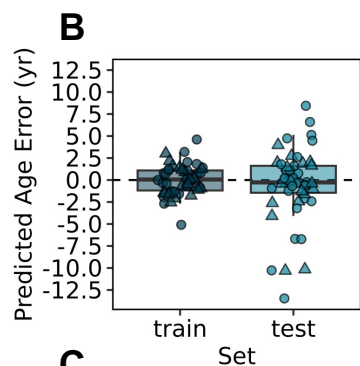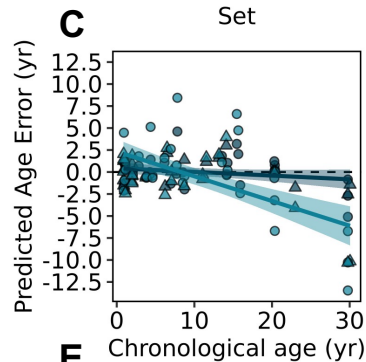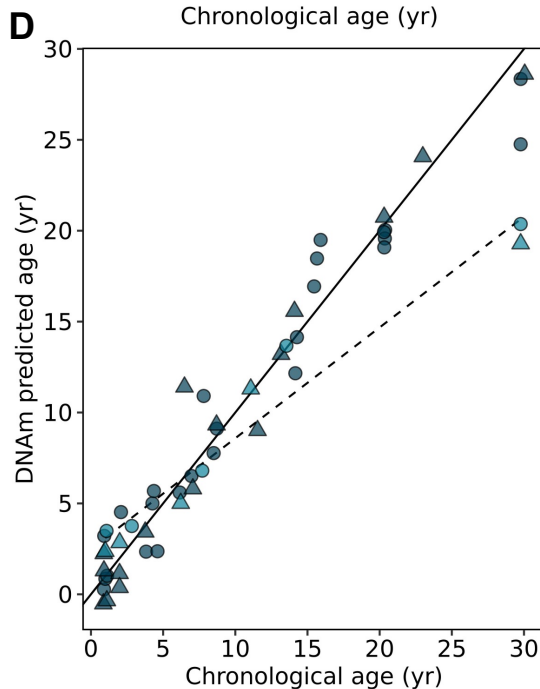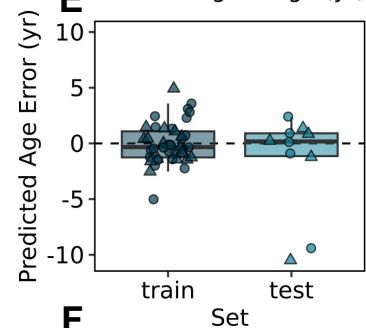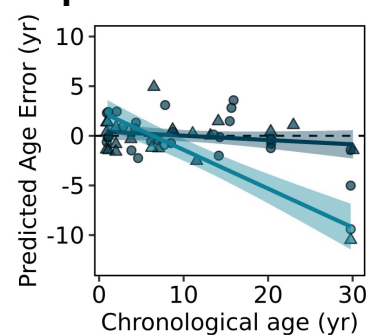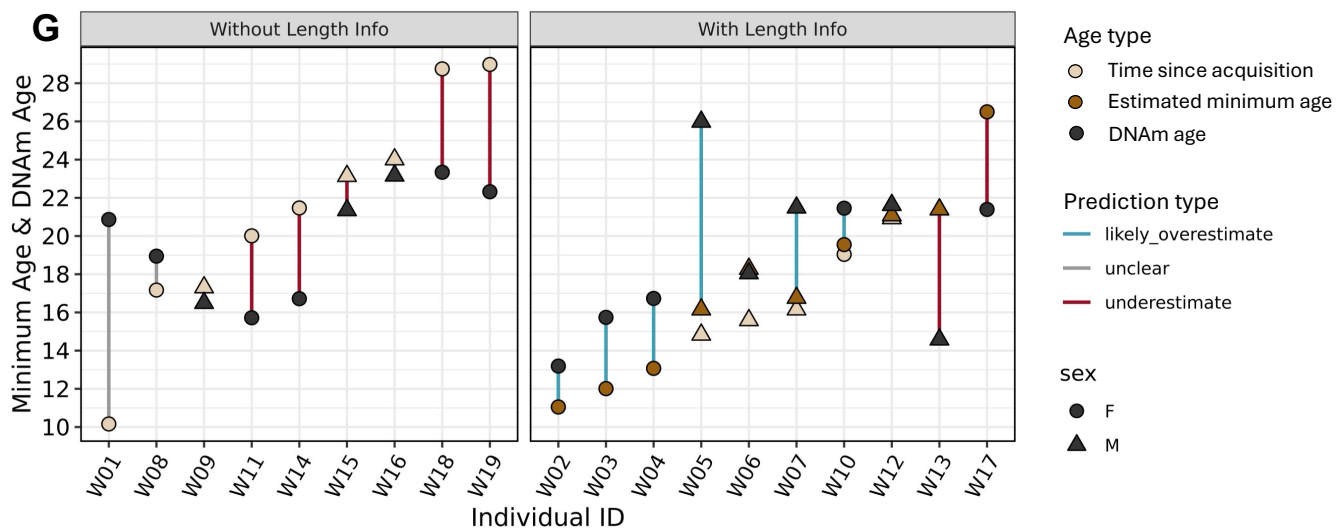

Supplement: Supplementary file 5 — Figure S4: Performance of epigenetic age estimators based on top 10 age‐associated CpGs in training set based on Pearson correlations. (A–C) Results from linear model calibrated via leave‐one‐out approach (D–F) Results from linear model calibrated with a single training and test set. (G) Results from linear model trained on all aquarium‐bred samples and tested on wild‐caught samples. [file MEC-35-e70326-s005.pdf]

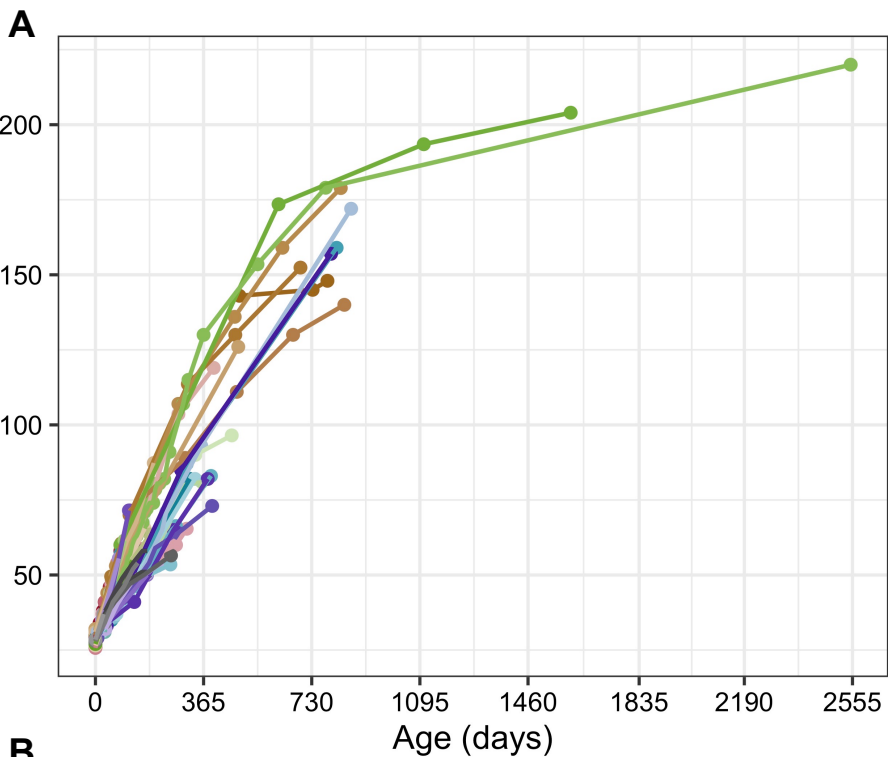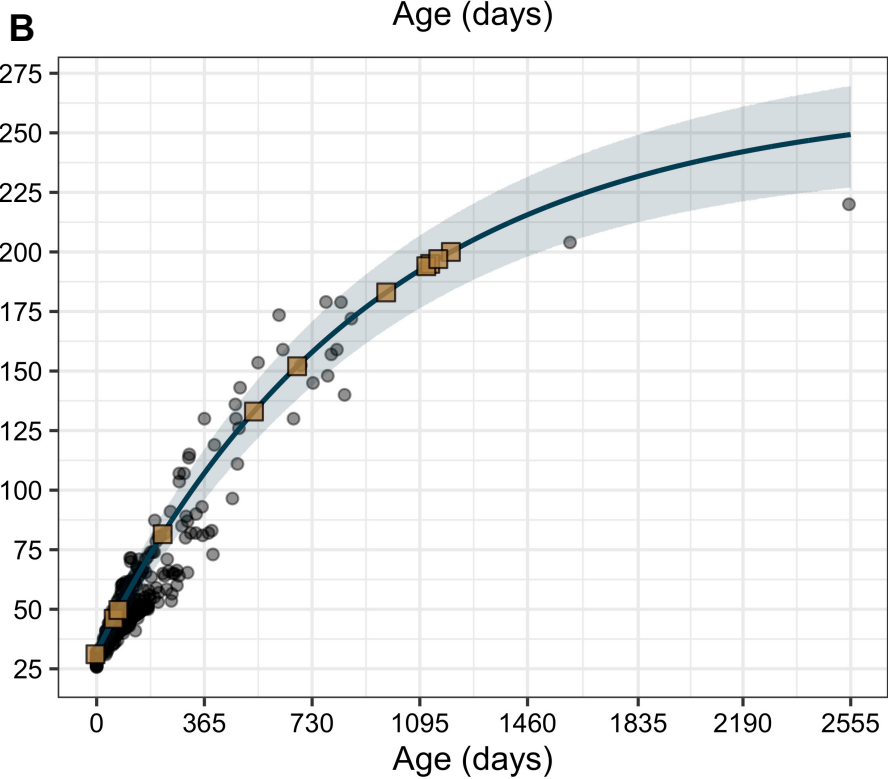

Supplement: Supplementary file 6 — Figure S5: Estimating age for wild‐caught zebra sharks. (A) Individual growth trajectories of 43 aquarium bred zebra sharks from 0 to 2555 days of age. (B) von Bertalanffy growth function fitted using non‐linear mixed effects model with random intercepts for individual ID. Ribbon around line indicates 95% prediction interval based on 10,000 simulations of fixed effect parameter sets according to the variance–covariance matrix of estimates. Brown squares indicate age predictions from total length for wild‐caught individuals calculated from inverse of von Bertalanffy growth function. [file MEC-35-e70326-s003.pdf]

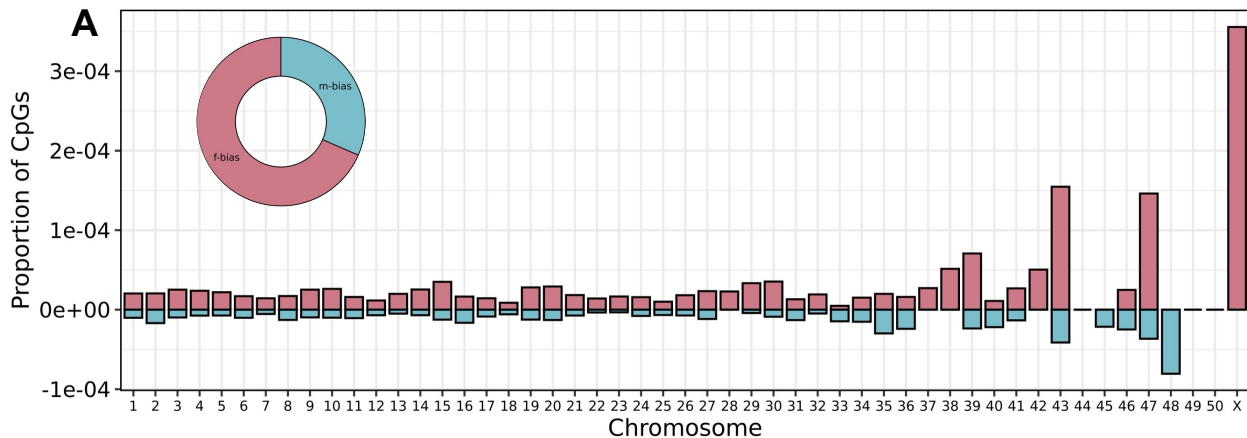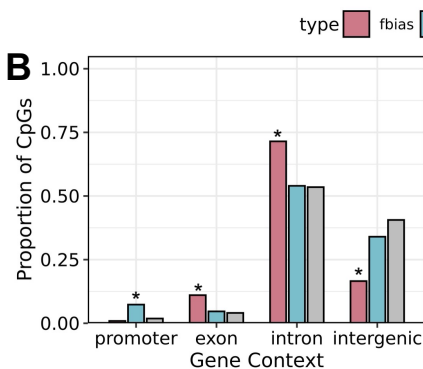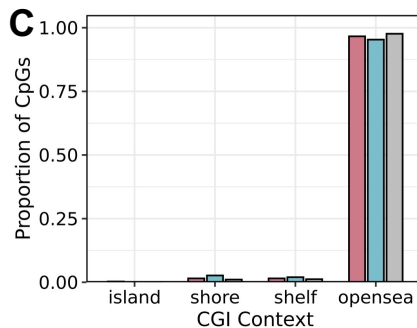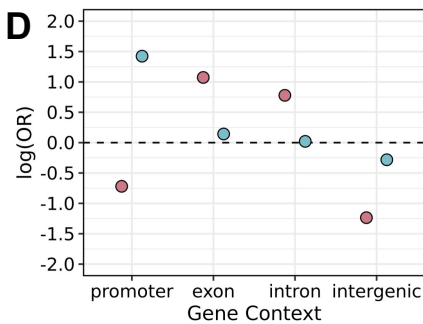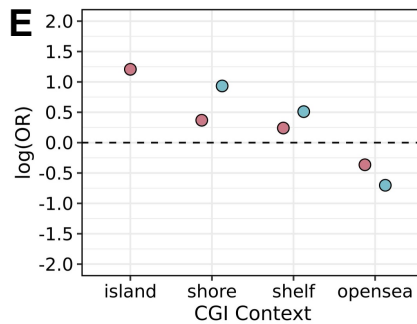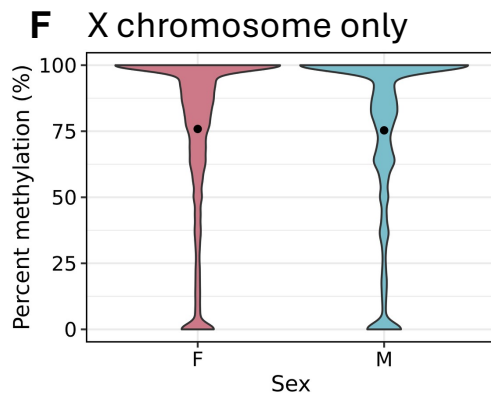

Supplement: Supplementary file 7 — Figure S6: Genome‐wide sex‐associated DNA methylation patterns. (A) Proportion of filtered CpGs per chromosome that are significantly differentially methylated between females and males (CpGs with female‐biased methylation in pink, CpGs with male‐biased methylation in blue). Inset donut plot depicts proportion of CpGs exhibiting differential methylation with respect to sex that show female biased methylation versus male‐biased methylation. (B, C) Proportion of sex‐associated CpGs overlapping different gene contexts and CpG island contexts. Grey bars depict proportion of all filtered CpGs that overlap with a given feature. Asterisks indicate a significant enrichment/depletion based on the results of a Fisher's exact test according to p < 0.05. (D, E) Log odds ratio of overlap with different gene or CpG island contexts. Values above zero indicate enrichment and values below zero indicate depletion. (F) Distribution of methylation status for all filtered CpGs located on the X chromosome in females and males. Central point indicates global mean. [file MEC-35-e70326-s008.pdf]
