# Supplementary material for: Genome‐Wide DNA Methylation Patterns Predict Age in the Zebra Shark (Stegostoma tigrinum) and Provide Insight Into the Evolution of Vertebrate Aging
Source: Mol Ecol. 2026 Apr 3;35(7):e70326. doi: 10.1111/mec.70326 (PMC13047888; doi:10.1111/mec.70326)
Supplement: Supplementary file 8 — Appendix S1: mec70326‐sup‐0008‐AppendixS1.pdf. [file MEC-35-e70326-s002.pdf]

Supplemental Information
Appendix S1. Supplementary Materials and Methods.

Optimization of elastic net regression hyperparameter alpha via leave-one-out cross validation

Elastic net penalized regression relies on the hyperparameter alpha to determine the balance between LASSO (L1) and ridge (L2) regularization (Zou & Hastie, 2005). An alpha value of 0 corresponds to strictly ridge regularization wherein predictor coefficients shrink towards zero, but none are assigned zero and therefore all predictors are included in the final model. An alpha value of 1 corresponds to strictly LASSO regularization wherein uninformative predictors are removed from the model and if a set of predictors are highly correlated with one another, the model tends to select only one of these predictors to be included in the model (Zou & Hastie, 2005). Epigenetic clocks have generally been fit with an alpha of 0.5 (Horvath, 2013), however, depending on the dataset, different alpha values may yield better performing models. As a preliminary test, we calibrated epigenetic clocks via leave-one-out cross validation for three different values of alpha – 0.2, 0.5, 0.8 – and compared the resulting performance according to median absolute errors and correlations (Pearson's r) between predicted ages and actual ages of aquarium-bred individuals. The results of this test are presented in the table below. Ultimately, we chose to use alpha = 0.5 because this yielded the best performing models in our test of multiple alpha values and is line with methods employed by previous studies.

Type	Alpha	Filtering	Weights	Test set correlation	Test set mean absolute error	Test set median absolute error
LOOCV (AB only)	0.2	none	none	0.966	2.017	1.148
	0.5	none	none	0.968	1.864	1.025
	0.8	none	none	0.962	1.928	1.211

Sequencing of repeated sample from zebra shark with anomalous DNAm predicted age

A total of 66 samples (48 aquarium bred, 18 wild-caught) was sequenced in the iteration of the study prior to generating the final sample set of 70 individuals. In the earlier iteration, a sample (GN22901) was sequenced from a wild-caught individual with a minimum age of 7.53 years. Records indicated this individual was acquired on May 30, 2013, and the first blood sample sequenced was taken on December 9, 2020. Based on the model trained on all 48 aquarium-bred individuals to predict raw age, GN22901 was predicted to be 25.50 years old (prediction error = 17.80 years). This represented the largest prediction error across all samples and models, prompting resampling of this individual to confirm this result and rule out technical errors. A second blood sample was taken from this individual on July 27, 2023 at a minimum age of 10.16 years and resulting DNA was sequenced (GN23051) according to the same previously described protocol. At this stage, we had generated the full dataset including all 70 individuals. A new model was trained on all 51 aquarium bred samples and tested on samples from the final 19 wild-caught individuals, including GN23051. In this case, GN23051 was predicted to be 20.86 years and this again represented the largest prediction error (10.70 years) of all samples. Based on this result, we concluded that this individual was likely acquired by the aquarium as an adult and minimum age (i.e., time since aquarium acquisition) was a substantial underestimate of true chronological age. Given that the aquarium at which this individual was housed did not have any records about size at acquisition, we were not able to generate a

corrected minimum age and thus we chose to present our model performance results with and without this individual. Only the second sample (GN23051) is included in the results.
